# Sulfidogenic Microbial Communities of the Uzen High-Temperature Oil Field in Kazakhstan

**DOI:** 10.3390/microorganisms9091818

**Published:** 2021-08-26

**Authors:** Diyana S. Sokolova, Ekaterina M. Semenova, Denis S. Grouzdev, Salimat K. Bidzhieva, Tamara L. Babich, Nataliya G. Loiko, Alexey P. Ershov, Vitaly V. Kadnikov, Alexey V. Beletsky, Andrey V. Mardanov, Nurlan S. Zhaparov, Tamara N. Nazina

**Affiliations:** 1 Winogradsky Institute of Microbiology, Research Center of Biotechnology of the Russian Academy of Sciences, 119071 Moscow, Russia; sokolovadiyana@gmail.com (D.S.S.); semenova_inmi@mail.ru (E.M.S.); salima.bidjieva@gmail.com (S.K.B.); microb101@yandex.ru (T.L.B.); loikonat@mail.ru (N.G.L.); e.alexey.mail@yandex.ru (A.P.E.); 2 SciBear OU, 10115 Tallinn, Estonia; denisgrouzdev@gmail.com; 3 Institute of Bioengineering, Research Center of Biotechnology of the Russian Academy of Sciences, 119071 Moscow, Russia; vkadnikov@bk.ru (V.V.K.); mortu@yandex.ru (A.V.B.); mardanov@biengi.ac.ru (A.V.M.); 4 Branch of the Limited Liability Partnership “KazMunaiGas Engineering”, Aktau 130000, Kazakhstan; zhaparov_n@kaznipi.kz

**Keywords:** oil field, microbial community, 16S rRNA, *dsrAB*, sulfate-reducing prokaryotes, biocide, nitrate

## Abstract

Application of seawater for secondary oil recovery stimulates the development of sulfidogenic bacteria in the oil field leading to microbially influenced corrosion of steel equipment, oil souring, and environmental issues. The aim of this work was to investigate potential sulfide producers in the high-temperature Uzen oil field (Republic of Kazakhstan) exploited with seawater flooding and the possibility of suppressing growth of sulfidogens in both planktonic and biofilm forms. Approaches used in the study included 16S rRNA and *dsrAB* gene sequencing, scanning electron microscopy, and culture-based techniques. Thermophilic hydrogenotrophic methanogens of the genus *Methanothermococcus* (phylum Euryarchaeota) predominated in water from the zone not affected by seawater flooding. Methanogens were accompanied by fermentative bacteria of the genera *Thermovirga*, *Defliviitoga*, *Geotoga*, and *Thermosipho* (phylum Thermotogae), which are potential thiosulfate- or/and sulfur-reducers. In the sulfate- and sulfide-rich formation water, the share of *Desulfonauticus* sulfate-reducing bacteria (SRB) increased. *Thermodesulforhabdus*, *Thermodesulfobacterium*, *Desulfotomaculum*, *Desulfovibrio*, and *Desulfoglaeba* were also detected. Mesophilic denitrifying bacteria of the genera *Marinobacter*, *Halomonas*, and *Pelobacter* inhabited the near-bottom zone of injection wells. Nitrate did not suppress sulfidogenesis in mesophilic enrichments because denitrifiers reduced nitrate to dinitrogen; however, thermophilic denitrifiers produced nitrite, an inhibitor of SRB. Enrichments and a pure culture *Desulfovibrio alaskensis* Kaz19 formed biofilms highly resistant to biocides. Our results suggest that seawater injection and temperature of the environment determine the composition and functional activity of prokaryotes in the Uzen oil field.

## 1. Introduction

Oil field microbial communities exist at a constant temperature under conditions of hindered water exchange; they do not depend on sunlight and the present-day atmosphere and may be considered as closed or semi-closed systems. In these ecosystems, oil is the main source of organic matter [1,2,3]. While crude oils are diverse in composition, qualitatively, oil consists of hydrocarbons and heterocyclic compounds containing oxygen, sulfur, nitrogen, and trace elements. Predominant components of oil gases are methane and related alkanes, CO_2_, and N_2_; H_2_ is less common, while H_2_S occurs in oil fields with sulfate-containing water. Amounts of molecular and ammonium nitrogen are sufficient to satisfy microbial growth requirements, while phosphorus content is low, limiting the microbial processes in these ecosystems. Nitrate and other nitrogen oxides do not occur in formation water. The possible electron acceptors in oil fields are CO_2_, sulfate and other oxidized sulfur compounds, or iron hydroxides. As a rule, oil fields do not contain dissolved oxygen. The processes of anaerobic degradation of oil organic compounds mediated by fermentative, syntrophic, sulfidogenic, and methanogenic prokaryotes are therefore of utmost importance [3,4,5]. The rates of microbial processes in oil fields are low due to low water exchange and limited amounts of biogenic elements and electron acceptors. 

Injection of coproduced water separated from oil and surface fresh water or seawater is used to maintain the stratal pressure in the course of oil field development. Application of sulfate-containing seawater activates sulfate reduction and results in sulfide accumulation in formation water, oil, and gas, which promotes the corrosion of steel equipment, increases the cost of oil production and processing, and causes environmental problems [6]. Quantitative assessment of the rates of anaerobic processes revealed the predominance of sulfate reduction in the degradation of oil organic matter in the oil fields with sulfate-containing waters (usually with carbonate collectors) and of methanogenesis, in low-sulfate oil fields (with sandstone collectors) [5,7,8,9,10]. Sulfate-reducing prokaryotes are considered the main sulfide producers and corrosion agents. However, thiosulfate-reducing bacteria were shown to occur in oil fields at higher number and may also be responsible for sulfide production [11,12]. The development of the methods to suppress growth of various groups of sulfidogenic bacteria is therefore an urgent task.

For the suppression of microbially influenced corrosion (MIC) and control of sulfide accumulation, injection of biocides is used [6]. However, biocide application requires significant expenses; moreover, formation of biofilms increases microbial resistance to biocides and thus prevents the suppression of sulfidogenesis [13,14]. Another way to control sulfide production is the injection of a nitrate solution, which stimulates the growth of heterotrophic denitrifying/nitrate-reducing bacteria and therefore nitrite release into formation water [14]. Nitrite inhibits the activity of sulfite reductase (DsrAB) and oxidizes sulfide, thereby decreasing the rate of sulfidogenesis and sulfide content in the reservoir, respectively. Cases of both successful and inefficient application of nitrate for sulfidogenesis suppression have been reported [15,16,17]. 

The Uzen high-temperature oil field at the Mangyshlak Peninsula, Kazakhstan, contains great amounts of high-paraffin oil; its highly saline formation water contains no sulfates. However, injection of the Caspian Sea water containing over 2 g·L^−1^ sulfates resulted in sulfate accumulation in the layers subjected to water-flooding [18]. 

The aim of this work was to determine the microbial community composition and potential agents of microbial corrosion in the injection and formation water of the Uzen oil field (Republic of Kazakhstan) and the possibility of suppressing the growth of sulfidogenic prokaryotes in planktonic and biofilm forms. 

## 2. Materials and Methods

### 2.1. Characteristics of the Petroleum Reservoir and Sampling Procedures

The Uzen oil field located in the western part of the Republic of Kazakhstan was studied. The XIII–XVIII Jurassic oil-bearing horizons are located at the depth of 1080–1370 m and have a temperature of 57–68 °C. According to analyses of surface samples, the density of crude oil was 0.8589 g·cm^−3^; it contained resin-paraffin components (22–28%) and had a high setting point of 28–32 °C. High temperature was maintained in the borehole in order to prevent paraffin precipitation. Oil viscosity was 16.5 mP·s at 50 °C. Coproduced gas contained methane (50.2%), ethane (19.8%), propane (16.8%), higher C_4_–C_5_ methane homologs (10.7%), N_2_ (2.3%), and CO_2_ (0.2%). Formation water of horizon XIV was of the calcium chloride type with a total salinity up to 120 g L^−1^ and did not contain sulfates. The Uzen oil field is exploited with water-flooding. Reservoir pressure is maintained by injection of coproduced water from the same deposit remaining after oil separation; this water was supplemented with the Caspian Sea water with a salinity 7–10 g L^−1^ [18].

Among the 21 water samples collected from the Uzen oil field were samples of formation water from 14 production wells, a sample of water from the near-bottom zone of injection well 2755 (backflush volume 30 m^3^), 3 samples of injection water represented by production water reinjected (PWRI) into the oil field from the first and the second injection stations and from petroleum oil treatment plant (separator) (PWRI-1, PWRI-2, and POTP), and 2 samples of Caspian Sea water collected at the inlet and outlet of the pumping station (SWin and SWout, respectively). Groundwater of the Alb-Cenomanian aquifer system of Cretaceous deposits widespread in the Southern Mangyshlak artesian basin was also sampled. For economic reasons, application of the Alb-Cenomanian water (AW) was proposed for injection, instead of the Caspian Sea water, which is delivered via a 150-km pipeline [19].

Water samples were collected on May 2019 at the wellhead of injection and production wells, dispensed into sterile bottles, hermetically sealed without air bubbles, and used at the day of sampling for the determination of abundance of cultivated microorganisms. The samples for chemical and molecular analyses were collected separately. The samples for chemical analyses were stored at 6 °C. Water samples (1 L each) for molecular studies were fixed with ethanol (1:1 *v/v*) at the day of sampling and filtered through 0.22-µm membranes (Millipore, United States). The filters were dried at room temperature and stored at −20 °C prior to analysis.

### 2.2. DNA Isolation, Amplification, and Sequencing of the 16S rRNA Gene

The composition of microbial communities from injection and formation water was determined by high-throughput sequencing of the 16S rRNA gene V3–V4 regions on the Illumina platform. Cell biomass obtained by filtration of ethanol-fixed water samples was washed off with the lysing solution containing 0.15 M NaCl and 0.1 M Na_2_EDTA (pH 8.0) and used for DNA isolation. Total DNA was isolated using the PowerSoil DNA Isolation Kit (MoBio, Carlsbad, CA, USA) according to the manufacturer’s recommendations and was stored at −20 °C. 

The purified DNA preparation was used as a template for PCR with a pair of primers to the V3–V4 regions of the 16S rRNA gene: 319F (5′-ACTCCTACGGGAGGCAGCAG-3′) and 806R (5′-GGACTACHVGGGTWTCTAAT-3) [20]. The primers were supplemented with oligonucleotide identifiers for sequencing on MiSeq (Illumina, San Diego, CA, USA). The 16S rRNA gene fragments were amplified using 5× Taq Red buffer and HS Taq polymerase (Evrogen, Moscow, Russia). The reaction mixture contained 5 µL of each primer (6 µM concentration), 5 µL DNA solution, and 15 µL PCR mix (1 U polymerase, 0.2 mM of each dNTP, and 2.5 mM Mg^2+^). DNA was amplified using the iCycler thermocycler model from Bio-Rad (Hercules, CA, USA). The PCR reaction conditions for DNA amplification were as recommended by Takahashi et al. [20]: initial denaturation at 98 °C for 2 min, followed by 35 cycles of annealing beginning at 65 °C and ending at 55 °C for 15 s, and elongation at 68 °C for 30 s. The annealing temperature was lowered 1 °C every cycle until reaching 55 °C, which was used for the remaining cycles. Fragments of the 16S rRNA gene were amplified on the template of DNA isolated from each specimen in three replicates, which were subsequently combined and purified by electrophoresis in a 2% agarose gel using a Cleanup Standard gel extraction kit (Evrogen, Moscow, Russia). High-throughput sequencing was performed using the MiSeq system (Illumina, San Diego, CA, USA) with a MiSeq Reagent Kit v3 (600 cycles) (Illumina) as recommended by the manufacturer. 

### 2.3. Bioinformatics Analysis

The obtained reads were further processed according to the workflow implementing suitable scripts from USEARCH version 10 [21]. Reads were demultiplexed (-fastx_demux), trimmed to remove the primer sequences (-fastx_truncate), and then quality filtered (-fastq_filter). UNOISE3 [22] was used to generate zero radius operational taxonomic units (zOTUs). zOTU is a term specific to analysis with UNOISE referring to operational taxonomic units which were generated by an error correction algorithm as opposed to a sequence similarity clustering algorithm [23]. Raw merged read pairs were mapped back to zOTUs using the -otutab command. zOTUs were submitted for taxonomic analysis in the RDP classifier [24]. Analysis of community composition using heatmaps was carried out using ClustVis [25]. 

### 2.4. Statistical Analysis and Functional Characterization

Statistical analysis was carried out using Microsoft Excel and Rstudio (the vegan package) [26]. Alpha-diversity and beta-diversity of the bacterial communities were estimated using the -alpha_div and -beta_div workflows, respectively. Beta-diversity of taxon abundance tables was visualized using non-metric multidimensional scaling (NMDS) of Bray–Curtis distances using the ‘metaMDS’ function in the R package Vegan version 2.5–6 (https://cran.ism.ac.jp/web/packages/vegan/vegan.pdf, (accessed on 1 September 2019)) [27].

The iVikodak software package [28] was applied to predict the functional characteristics of bacterial communities, using phylogenetic analysis of the 16S amplicon data generated from the studied samples. The list of genera for all 16S rRNA gene libraries based on the OTUs table (% of total reads for each library) was provided as input in iVikodak. The Global Mapper module was used for inferring the functional profiles and Local Mapper was used for prediction of the profiles of the individual pathway enzymes using the KEGG pathway database. Pathway exclusion cut-off (PEC) was selected as set at 80 to strengthen the confidence of data. Relevant heatmaps based on the results of the metabolic inference on the genus level were constructed using the ClustVis [25] online resource (http://biit.cs.ut.ee/clustvis/ (accessed on 8 April 2021)). 

### 2.5. dsrA Illumina Sequencing, Bioinformatics Processing, and Data Analyses

Barcoded *dsrA* amplicons for Illumina sequencing were prepared from extracted DNA by using the two-step PCR protocol (Illumina). In the first PCR, an approximately 440-bp fragment of the *dsrA* gene was amplified with the primer set DSR-1Fdeg/PJdsr853Rdeg including the Illumina sequencing adapters [29]. The first-step PCR program was performed with an initial denaturation at 96 °C for 3 min, followed by 30 cycles of 30-s denaturation at 96 °C, 30-s annealing at 56 °C and elongation for 1 min at 72 °C, and a final elongation step at 72 °C for 10 min. For the second PCR, dual indices were attached during eight cycles using the Nextera XT Index Kit. Paired-end sequencing was performed in a MiSeq platform (Illumina, San Diego, CA, USA) with a MiSeq Reagent Kit V3 (2 × 300 cycles). The overlapping Illumina read pairs were merged into longer reads using FLASH v1.2.11 [30]. Merged reads were compared to the uniref protein database [31] using diamond v2.0.6.144 [32], and the reads that had a significant protein alignment (evalue > 1 × 10^−3^) were used for further analysis. Low-quality reads were removed using USEARCH v11, high-quality reads were clustered into OTUs with 97% identity threshold, chimeras and singletons were removed during a clustering process by the USEARCH algorithm. All initial reads (including low-quality reads and singletons) were mapped back to OTU representative sequences at minimum 97% identity to estimate OTUs size for each sample.

### 2.6. Media Composition

Microbial numbers were determined by inoculating tenfold dilutions of the water samples into liquid media in two replicates as described earlier [33]. The results were calculated using the McCready tables of the most probable numbers. The basal medium for enumeration of all microbial groups was marine mineral medium (MM) containing per liter distilled water, 3.0 g MgCl_2_·6H_2_O, 0.3 g KCl, 0.15 g CaCl_2_·2H_2_O, 0.3 g NH_4_Cl, 0.2 g KH_2_PO_4_, 20.0 g NaCl, and 2.5 g NaHCO_3_ [34]; and trace elements [35], 1 mL. For enumeration of aerobic bacteria, the medium was supplemented with glucose (1.0 g L^−1^), bacto-tryptone (5.0 g L^−1^), and yeast extract (2.5 g L^−1^), at pH 7.0–7.2, with air as the gas phase. The medium for fermentative bacteria contained per liter mineral medium, 4.0 g peptone, 10.0 g glucose, and 0.5 g Mohr’s salt (FeSO_4_·(NH_4_)_2_SO_4_·6H_2_O), at pH 6.5–7.0, with argon as the gas phase [36]. Sulfate-reducing (SR) prokaryotes were assessed by sulfide production in the terminal dilutions in MM medium supplemented with Na_2_SO_4_ (4.0 g L^−1^), FeSO_4_·7H_2_O (0.01 g L^−1^), yeast extract (0.5 g L^−1^), Na_2_S·9H_2_O (0.1 g L^−1^), and sodium lactate (4.0 g L^−1^), at pH 7.0–7.2, with argon as the gas phase. Methanogens were assessed by methane accumulation in MM medium supplemented with sodium acetate (2.5 g L^−1^), methanol (2 mL L^−1^), yeast extract (1.0 g L^−1^), and Na_2_S·9H_2_O (0.5 g L^−1^), at pH 7.0–7.2; with H_2_/CO_2_ (4:1) as the gas phase. Enrichment cultures of thiosulfate- (TS) and sulfur-reducing bacteria were obtained in the medium for fermentative bacteria supplemented with electron acceptors: thiosulfate Na_2_S_2_O_3_·5H_2_O (2.0 g L^−1^) or sulfur (2.0 g L^−1^); Mohr’s salt was replaced by FeSO_4_·7H_2_O (0.01 g L^−1^). Enrichment cultures of denitrifying/nitrate-reducing bacteria (DNB) were obtained in MM medium supplemented with NaNO_3_ (1.0 g L^−1^) and sodium acetate (2.5 g L^−1^); at pH 7.0–7.2, with argon as the gas phase. Anaerobic growth of nitrate-reducing/denitrifying bacteria was monitored by changes in nitrate and nitrite concentrations in the medium, and of nitric oxide and molecular nitrogen in the gas phase. 

Depending on the temperature of the environment used for sampling, vials inoculated with Kaspian Sea water (SWin and SWout) were incubated at 25 °C; vials inoculated with the samples of production water reinjected into the oil field from injection stations 1 and 2 (PWRI-1 and PWRI-2) and from the station of petroleum oil treatment (separation) plant (POTP) were incubated at 42 °C; samples of formation water from production wells and of Alb-Cenomanian water were incubated at 55 °C. Incubation was carried out for 14 days; absence of growth was registered after 30 days of incubation.

To determine the effect of nitrate on sulfide production by sulfate- (SR) and thiosulfate-reducing (TSR) microorganisms from formation and injection water, Ca nitrate in concentrations of 0, 0.5, 1.0, 1.5, and 2.0 g L^−1^ (calculated for the nitrate ion) was added to MM medium supplemented with yeast extract (0.5 g L^−1^), sodium lactate (2 g L^−1^), and sucrose (5 g L^−1^). Electron acceptors for SR and TSR bacteria were Na_2_SO_4_ (2.8 g L^−1^) and Na_2_S_2_O_3_·5H_2_O (1.6 g L^−1^), respectively. The medium was reduced with Na_2_S·9H_2_O (0.05 g L^−1^). Argon was used as the gas phase. 

### 2.7. Analytical Techniques and Biofilms Formation

Methane, hydrogen, nitrogen, and CO_2_ in the headspace were determined by gas chromatography. Sulfide was determined colorimetrically with *p*-phenylenediamine by the method described by Trüper and Schlegel [37]. The chemical composition of formation water was determined as described elsewhere [8]. Volatile fatty acids and lower alcohols were analyzed on a GC-2010 Plus gas chromatograph (Shimadzu, Kyoto, Japan) [38]. The nitrate ion concentration was determined using an ion-selective nitrate electrode and an Expert-001 ionometer (Econix-Expert, Moscow, Russia). Nitrite ion concentration was measured using Quantofix test strips (Macherey-Nagel, Düren, Germany). 

Sulfate-reducing bacterium *Desulfovibrio alaskensis* strain Kaz19 was used for determination of the effect of biocides on sulfide formation and survivability of planktonic and biofilm forms. For this purpose, a mineral carrier (steel 20 coupon) and glutaraldehyde or Rancid 7000–7020 (RuanNalco Co., http://rauannalco.kz/produkciya/bakteritsidy (accessed on 5 March 2021)), a biocide widely used at the oil fields in Kazakhstan, were added to the culture media. The glutaraldehyde concentrations used were 0 (Control), 100, 250, and 500 mg L^−1^; Rancid was used at concentrations 0 (Control), 40 (minimal work concentration), 80, and 120 mg L^−1^ to compare the effectiveness of its impact. The initial cell number in inoculated cultures was 2.5 × 10^7^ cells·mL^−1^. The cultures were incubated for 14 days at 30 °C. Sulfide formation was monitored during the incubation. Biofilms formation on the mineral carrier and growth of planktonic cultures were determined by staining with MTT (3-(4,5-dimethylthiazol-2-yl)-2,5-diphenyltetrazolium bromide) with subsequent extraction of produced formazan by dimethyl sulfoxide (DMSO) and measuring the extract optical density at 500‒600 nm, as was proposed previously [39,40].

### 2.8. Microscopic Techniques

To obtain scanning electron microscopy images of biofilm growth, enrichment cultures were grown in the relevant liquid media with Teflon cubes; then the medium was decanted and the biomass was washed twice off the Teflon cubes with phosphate buffer (pH 7.0), collected, and dehydrated in ethyl alcohol solutions of increasing concentration (from 15 to 100%). Then, the samples were washed twice with 100% acetone and dried at the critical point using a special chamber. The dried preparations were mounted on special tables, and then a thin layer of metal (gold/palladium) was sprayed onto the preparations to create a conductive coating. The samples obtained were examined under a Camscan-S2 scanning electron microscope (Cambridge, UK) (accelerating voltage 20 kV, SEI mode) and under a TM3000 scanning electron microscope (Hitachi, Tokyo, Japan) (accelerating voltage 15 kV). The research was carried out using the equipment purchased under the Moscow State University Development Program.

### 2.9. Nucleotide Sequence Accession Numbers

The GenBank/EMBL/DDBJ accession number of the 16S rRNA gene sequence of *Desulfovibrio alaskensis* strain Kaz19 is MZ604343. The raw data generated from 16S rRNA and *dsrAB* gene sequencing of microbial communities from injection and production water samples have been deposited in the NCBI Sequence Read Archive (SRA) under the accession numbers SRR15234354–SRR15234380 (Bioproject PRJNA749317) and SRR15293245, SRR15293278–SRR15293283 (Bioproject PRJNA749166), respectively.

## 3. Results

### 3.1. Environmental Characterization and Cultivable Microorganisms of the Injection and Production Water Samples

The original formation water of horizon XIV had high total salinity (up to 120 g L^−1^), with predominance of sodium, calcium, and magnesium chlorides, and with almost complete absence of sulfates. No sulfates were detected in 8 out of the 14 water samples from production wells; sulfide concentration in these 8 samples was in a range of 2.4–91.0 mg L^−1^, while salinity varied from 24.2 to 80.5 g L^−1^ (Table 1). Water samples from the remaining 6 production wells (6928, 9064, 8533, 6916, 7574, and 3239), from the near-bottom zone of an injection well 2755 (30 m^3^), from the injection stations 1 and 2 (PWRI-1 and PWRI-2), and from the petroleum oil treatment plant (POTP) consisted of mixtures of formation water and seawater with salinity in a range of 14.0–120.5 g L^−1^ and sulfate concentrations from 19.8 to 1281.7 mg L^−1^. Most production water samples had pH 6.2–7.0. While both seawater (SWin and SWout) and the Alb-Cenomanian water (AW) were similar in salinity (9.6–12.8 g L^−1^), had high concentrations of sulfates (2181.5–3161.9 mg L^−1^), and almost did not contain sulfide; seawater had higher concentrations of calcium and magnesium ions than Alb-Cenomanian water. 

Abundance of cultivable microorganisms belonging to the major physiological groups (aerobic organotrophic, anaerobic fermentative, sulfate-reducing, and methanogenic prokaryotes) was determined. In the water from the near-bottom zone of an injection well 2755 (backflushed volume 30 m^3^), and in water samples from PWRI-1, PWRI-2, and POTP, the number of mesophilic microorganisms (cultivated at 42 °C) was considerably higher than abundance of thermophilic microorganisms (cultivated at 55 °C) in samples of production water (Figure S1). The most numerous populations of fermentative bacteria were revealed in the samples of injection and formation water. Dinitrogen production by heterotrophic denitrifying bacteria was observed in the cultures from seven water samples (PWRI-1, PWRI-2, SWin, SWout, POTP, 3239, AW, and 2755-30); nitrite formation was detected in the cultures from six water samples (9064, 9677, POTP, 6038, 3239, and 2755-30).

In 10 out of 14 production water samples, sulfate-reducing prokaryotes (enumerated in the medium with lactate as the carbon and energy source) were not found. Sulfide formation was, however, observed in 10 and 12 cultures from production wells grown in rich medium for fermentative bacteria containing peptone and glucose and supplemented with sulfur or thiosulfate, respectively (Figure S2). 

### 3.2. Microbial Diversity in Petroleum Reservoir Based on 16S rRNA and dsrA Gene Gequencing

From 21 water samples were generated 1,482,093 high-quality reads of the V3–V4 region 16S rRNA gene (Table S1). The libraries contained from 34 to 655 OTUs with 97% sequence similarity within OTU. The highest OTU numbers were recovered in the libraries from production waters reinjected into the oil field (PWRI-1, PWRI-2) and from the water of production well 8533. Non-parametric indicators of diversity showed similar trends, with the highest Simpson indexes in these libraries. 

**Figure 1:**
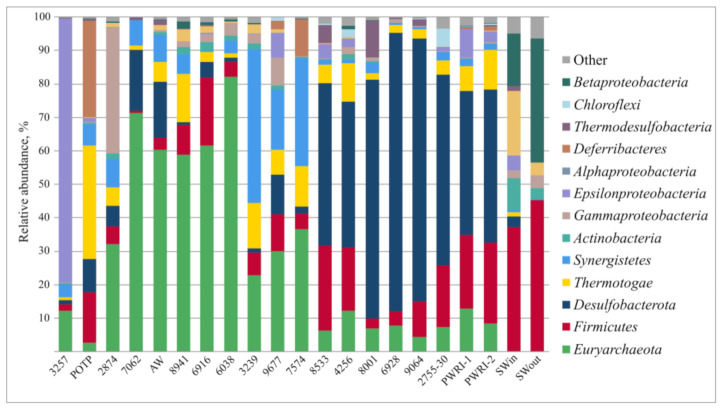
Taxonomic classification of archaeal and bacterial 16S rRNA gene fragments in the libraries from injection and production water samples from the Uzen oil field at the phylum level (at the class level for *Proteobacteria*) using the RDP classifier. The taxa constituting at least >1% in each library are listed.

Water samples collected at various sites along the production lines exhibited differences in microbial community composition. Both bacteria and archaea were present in formation water samples, while injected Caspian seawater (SWin and SWout) contained only members of the bacteria domain (Figure S3). The share of archaeal 16S rRNA gene sequences in the libraries from the oil field waters varied from 2.6% to 82.1% (Figure 1). 

**Figure 2:**
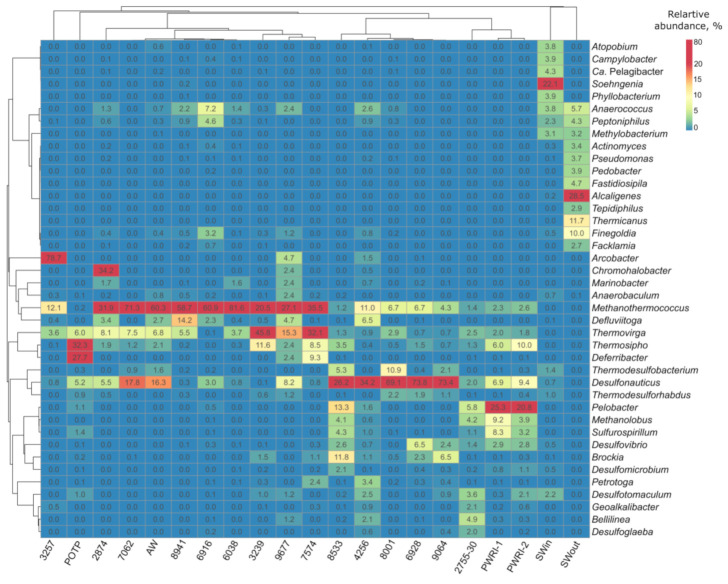
Heatmap of the 40 genera with the highest relative abundance in the libraries of the 16S rRNA gene sequences from injection and production water samples from the Uzen oil field. Representation of the genus was calculated as sequence proportions divided by total sequence count in each library. Columns are clustered using correlation distance and average linkage.

The majority of bacterial OTUs detected in the libraries of production water samples were affiliated with Firmicutes, Desulfobacterota, Thermotogae, and Sinergistetes (Figure 1). Most archaeal sequences of the phylum Euryarchaeota belonged to thermophilic hydrogenotrophic methanogens of the genus *Methanothermococcus*, which were revealed in the water from all studied production wells (Figure 2). Sulfate-reducing archaea of the genus *Archaeoglobus* were found in four samples; their share in the libraries did not exceed 0.33%.

Predominant phyla/classes in the water from injection well (2755-30) were Desulfobacterota (55.7%), Firmicutes (18.0%), Chloroflexi (5.3%), and Thermotogae (4.1%), as well as Euryarchaeota archaea (7.3%). This community comprised diverse thermophilic and mesophilic sulfate-reducing bacteria of the genera *Desulfotomaculum* (3.5%), *Desulfonauticus* (2.0%), *Desulfovibrio* (1.25%), and *Desulfoglaeba* (2.0%), as well as fermentative bacteria of the genus *Thermovirga* (2.5%), which are able to reduce sulfur to sulfide.

The microbial community of the water sample POTP included bacteria Thermotogae (33.5%), Deferribacteres (28.5%), Firmicutes (14.9%), Desulfobacterota (9.8%), and Synergistetes (6.2%); and archaea of the phylum Euryarchaeota (2.6%).

The microorganisms of injected seawater belonged mainly to the classes Beta- and Alphaproteobacteria, and to the phyla Firmicutes and Actinobacteria. Seawater samples (SWin and SWout) contained a number of bacteria uncommon in oil fields, including members of the genera *Alcaligenes*, *Thermicanus*, *Finegoldia*, and *Peptoniphilus*, as well as marine alphaproteobacteria *Candidatus* Pelagibacter (Figure 2). Sulfate-reducing bacteria of the genera *Desulfotomaculum*, *Thermodesulfobacterium*, *Thermodesulforhabdus*, *Desulfonauticus*, *Desulfomicrobium*, and *Desulfovibrio* were delivered with seawater (about 6.3% of sequences in SWin).

**Figure 3:**
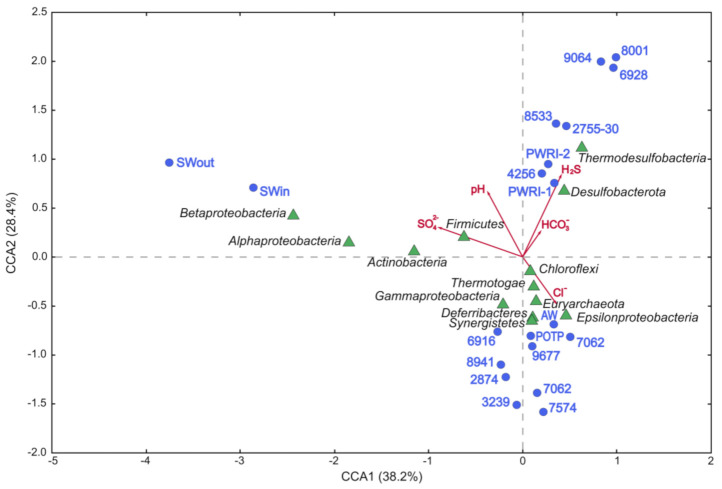
Canonical correlation analysis showing the correlation between the geochemical parameters and prokaryotic diversity at the phylum (class) level in the 16S rRNA gene libraries from microbial communities of seawater and formation water from the Uzen oil field.

Canonical correlation analysis (CCA) revealed that microbial communities fell into three groups (Figure 3). The first group contained the communities of production water samples with low sulfate and sulfide concentrations and of the subterranean community of the Alb-Cenomanian water (AW). The second group contained the communities from production wells (6928, 9064, and 8533) with high levels of sulfide and sulfates, as well as similar communities 2755-30, PWRI-1, PWRI-2, and POTP. The third group formed the communities of injection seawater (SWin and SWout), which differed significantly from the oil field communities. 

Dependence of the composition of microbial communities on the geochemical parameters of their habitats was revealed. Correlations were found between high sulfide concentrations and occurrence of members of the classes Thermodesulfobacteria and Desulfobacterota, which comprise most taxa of sulfate-reducing bacteria, and with high concentrations of bicarbonate, a product of oil biodegradation. Methanogenic archaea of the phylum Euryarchaeota and fermentative bacteria of the classes Thermotogae and Synergistia were associated with oil field zones, where original formation water contained zero or low sulfate and sulfide concentrations. These results are in agreement with the data on the relationship between microbial diversity and such environmental parameters of oil fields as temperature, water-flooding, salinity, and sulfate and sulfide content, which were previously reviewed by Li et al. [41].

Gene libraries for *dsrA* (coding dissimilatory sulfite reductase α-subunit) were constructed from seven injection and production water samples (Figure S4a,b). Certain obtained sequences were similar to *dsrA* found in members of 15 genera, including *Desulfonauticus*, *Desulfovibrio*, *Desulfomicrobium*, *Desulfotomaculum*, *Desulfoglaeba*, and *Moorella*, as well as novel sequences belonging to uncultured bacteria from a high-temperature oil field [17].

Moreover, molecular surveys using 16S rRNA and *dsrAB* gene sequencing revealed sulfate-reducing bacteria in production water samples, where they were not detected by culture-based techniques.

### 3.3. Potential Functional Characteristics of Microbial Communities of Injection and Production Water Samples from the Uzen Oil Field

**Figure 4:**
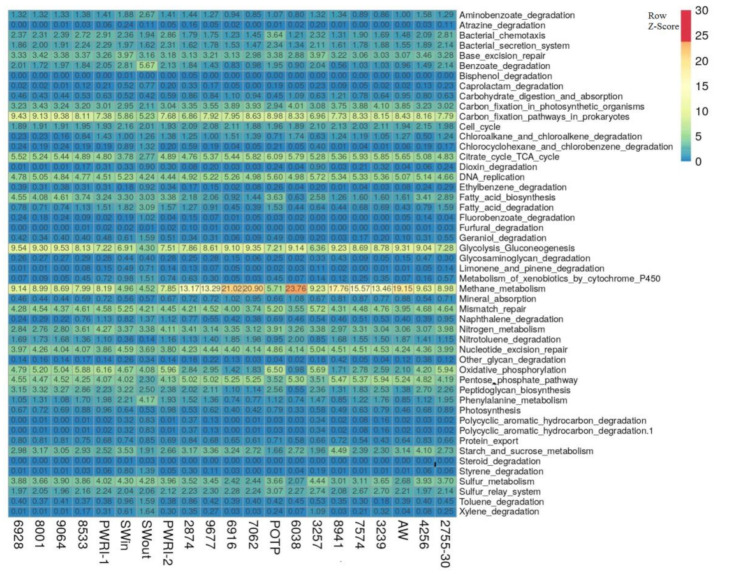
The heatmap showing the clustering of injection and production water samples from the Uzen oil field based on the relative percentage of iVikodak-derived microbial community functional profiles.

The functional characteristics of the microorganisms were predicted using the Global Mapper module of the iVikodak software based on the KEGG database. Based on the relationship between phylogeny and function, the iVikodak platform allows predicting potential functional community activity using 16S rRNA data [28]. The studied communities mainly carried out the pathways of methane, nitrogen, and sulfur metabolism, carbon fixation, glycolysis/gluconeogenesis, citrate cycle, and benzoate degradation (Figure 4).

Archaea of the genus *Methanothermococcus* had the greatest functional potential for methane, nitrogen, and sulfur metabolism pathways in the samples of production water where they were predominant (6916, 7062, 8941, Figure S5a–c). Bacteria of the genera *Desulfonauticus*, *Desulfotomaculum*, *Thermodesulfobacterium*, and *Desulfoglaeba* mainly contributed to the sulfate reduction process (Figure S5b). Bacteria of the genera *Pelobacter*, *Sulfurospirillum*, and *Deferribacter* occurred mainly in the zones with alternating oxic-anoxic conditions (2755-30, PWRI-1, PWRI-2, POTP), where they were involved in the metabolism of nitrogen and sulfur compounds (Figure S5b,c). Predicted enzyme profiles for methane metabolism, sulfur metabolism, and nitrogen metabolism pathways of microorganisms in the samples from the near-bottom zone of injection well 2755-30 and from production well 7062 are presented in Figures S6 and S7. These data indicate higher potential activity of denitrifying population in the near-bottom zone of injection well 2755 (30 m^3^) in comparison with the zone of production well 7062 (Figures S6c and S7c).

Therefore, our results corroborate the hypothesis that while the microbial communities of the Uzen oil field are mainly involved in methanogenic oil degradation, injection of sulfate-containing seawater or the planned injection of Alb-Cenomanian water may stimulate the undesired process of sulfate reduction. The thiosilfate- and sulfur-reducing population of fermentative bacteria *Thermovirga*, *Defluviitoga*, and *Petrotoga* may also be able to contribute to sulfidogenesis.

### 3.4. Effect of Nitrate Addition on Sulfide Production by SR and TSR Enrichments from the Oil Field

Nitrate injection into oil reservoirs in order to suppress development of sulfate-reducing bacteria with nitrite, a product of nitrate reduction, is presently gaining popularity [15,42]. We studied the effect of different nitrite concentrations on sulfide formation by sulfate- and thiosulfate-reducing bacterial populations from the Uzen oil field. Sulfide production by all studied thermophilic and mesophilic TSR and by several SR cultures was registered in the medium containing no nitrate (Figure S8a). Amendment of the medium with 0.5–1.5 g NO_3_^−^ L^−1^ resulted in decreased sulfide production, especially in thermophilic TSR (9677, 3239) and SR (AW, POTP) enrichments, where nitrite accumulated up to 100 mg L^−1^ (Figure S8b). However, mesophilic TSR and SR populations from the zone of injection well 2755 (30 m^3^) were not sensitive to nitrate concentrations up to 2 g NO_3_^−^ L^−1^. Nitrite was not detected in these cultures and nitrate was completely reduced to dinitrogen by denitrifying bacteria. 

### 3.5. Molecular Analyses of Sulfate-, Thiosulfate-, and Nitrate-Reducing Enrichments

Enrichment cultures of sulfate-reducing, thiosulfate-reducing, and denitrifying microorganisms were studied in order to determine why sulfidogens were not suppressed by nitrite, a metabolite of denitrifying bacteria. Since nitrate is usually introduced into oil reservoirs with injected water, the composition of enrichments from the near-bottom zone of an injection well 2755 (30 m^3^) was studied. Enrichments from the petroleum oil treatment plant (POTP, separator), containing a mixture of formation water from a number of producing wells, were studied for comparison. 

The 16S rRNA gene libraries from the enrichments contained 59,710 to 90,139 nucleotide sequences, which formed 72 to 322 OTUs and exhibited high Good’s coverage values (Table S2). Using the media with various electron acceptors resulted in the detection of prokaryotes poorly represented in the original formation water. No archaea were found in the SR, TSR, and DNB enrichments from 2755-30. The ratios of higher bacterial taxa in SR-2755-30 and TSR-2755-30 enrichments were different from the DNB-2755-30 denitrifying community (Figure S9A). Application of sulfate- or thiosulfate-containing media enriched with organic substrates resulted in preferential development of sulfidogenic bacteria of the genera *Desulfovibrio*, *Geotoga*, and *Thermovirga*, which are adapted to such conditions and substrates. The OTUs of SRB detected in the enrichments shared 100% sequence identity (450 nt) with *Desulfovibrio alaskensis* [43] and *Desulfonauticus autotrophicus* [44] isolated from similar habitats and with *Desulfomicrobium thermophilum* isolated from a terrestrial hot spring [45]. The denitrifying enrichment DNB-2755-30 contained bacteria of the genera *Marinobacter*, *Sulfurospirillum*, and *Halomonas*, reducing nitrate, and fermentative bacteria of the genera *Acidaminobacter* and *Geotoga*, incapable of nitrate reduction.

In the SR-POTP sulfate-reducing enrichment, comprising bacteria of the genera *Thermosipho*, *Desulfovibrio*, and *Geotoga*, only members of *Desulfovibrio* were capable of reducing sulfate to sulfide (Figure S9B). In the thiosulfate-reducing population (TSR-POTP), uncultured bacteria (FN356255) predominated, which have been earlier detected in the seawater-injected Dan high-temperature oil field [17], and *Thermovirga* spp. In the DNB-POTP enrichment, bacteria of the genera *Marinobacter*, *Thermus*, and *Pseudomonas* were detected, which are capable of growth both as aerobic hydrocarbon-oxidizing organotrophs and as denitrifiers. Thus, despite the absence of nitrate in injection and production water, mesophilic and thermophilic bacteria potentially capable of nitrate reduction to dinitrogen inhabited the Uzen oil field.

### 3.6. Effect of Biocides on Survival of Planktonic Cells and Biofilms of Desulfovibrio alaskensis Kaz19 and on Sulfide Production

**Figure 5:**
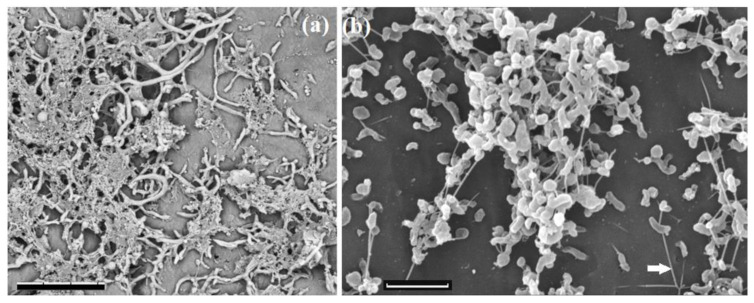
Scanning electron micrographs of biofilms of a sulfate-reducing enrichment obtained from the near-bottom zone of injection well (**a**) and of the *Desulfovibrio alaskensis* Kaz19 (**b**) grown in lactate-sulfate medium for 14 days. Morphological diversity of cells embedded into exopolymers in biofilms-producing enrichment culture (**a**) and pilus-like nanowires which connect the cells of *D. alaskensis* Kaz19 (**b**) can be seen. The images were obtained under a scanning electron microscope TM3000 (Hitachi, Japan) (accelerating voltage 15 kV) (**a**) and under a scanning electron microscope Camscan-S2 (Cambridge, UK) (accelerating voltage 20 kV, SEI mode) (**b**). Arrow indicates the pilus-type nanowires that touch both the surface and other cells. Scale bars, 10 µm (**a**), 3 µm (**b**).

The pure culture, strain Kaz19, was isolated from the sulfate-reducing enrichment 2755-30 by successive transfer from liquid to solid marine lactate-sulfate MM medium with 2.0% NaCl (wt/vol). Since the 16S rRNA gene sequence of the strain Kaz19 (accession number MZ604343) had 100% similarity with that of *Desulfovibrio alaskensis* strain Al1^T^ [43], the new strain Kaz19 was assigned to this species. Cells of the sulfate-reducing strain Kaz19 were vibrio-shaped, with a single polar flagella. The strain utilized lactate and pyruvate as energy and carbon sources, reducing sulfate, sulfite, and thiosulfate to sulfide. In a medium with molecular hydrogen, acetate, and sulfate wall growth occurred, resulting in biofilm formation. The strain Kaz19 grew at 30 °C. Both the SR-2755-30 enrichment and the pure culture of *D. alaskensis* Kaz19 grew as biofilms (Figure 5a,b). This strain was used for determination of the effect of biocides on sulfide formation and survivability of planktonic and biofilm forms. 

Sulfide was detected in the control medium without biocides (309 mg S^2−^·L^−1^) and in the cultures grown with Rancid at all studied concentrations (40, 80, and 120 mg L^−1^), but not in the cultures with glutaraldehyde (Figure S10a). After 14 days of cultivation, the cell cultures were separated into planktonic and biofilm fractions, and the percentage of surviving cells was determined by staining with MTT in comparison with the control sample without biocide addition. The results of the experiment demonstrated that glutaraldehyde suppressed both sulfide production by strain Kaz19 and its planktonic growth, while biofilms were not sensitive to glutaraldehyde (Figure S10b). Rancid concentrations used in our experiments did not suppress survival of *D. alaskensis* strain Kaz19 and sulfide production. 

## 4. Discussion

The Uzen oil field is characterized by high stratum temperature (57–68 °C) and formation water salinity (up to 120 g L^−1^), with no sulfate present in the original formation water. Using the Caspian Sea water to maintain the formation pressure resulted in a temperature decrease in the near-bottom zone of injection well to 40–45 °C and in emergence of sulfate and sulfide in the layers subjected to flooding. To detect sulfide producers in the Uzen oil field, 21 water samples were collected from various sites along the production lines and investigated using physicochemical and culture-based techniques and by 16S rRNA and *dsrAB* gene sequencing. Physiological properties of the microorganisms detected in the oil field were in agreement with the high temperature and salinity of this habitat.

Predominant phyla in the water from production wells were Euryarchaeota, Firmicutes, Desulfobacterota, Thermotogae, and Sinergistetes (Figure 1 and Figure 2, and Figure S3). These patterns of phylum-level distribution were similar to those of other oil fields with high-salinity formation water exploited with seawater-flooding [16].

In the original sulfate-free formation water (7062, 2874, 8941, 6038), thermophilic hydrogenotrophic methanogens of the genus *Methanothermococcus* (phylum Euryarchaeota) were predominant (Figure 2 and Figure 3). Sulfate-reducing archaea of the genus *Archaeoglobus* were among the minor components (below 0.33% of the sequences in four libraries). Methanogens detected in the Uzen oil field, as well as those isolated from the North Sea oil fields [46] and near the California coast [47], were related to the type strain *Methanothermococcus* (previously *Methanococcus*) *thermolithotrophicus* SN-1^T^ [48]. Takai and co-workers [49] suggested that hyperthermophilic and thermophilic methanogens of the order *Methanococcales* are cosmopolitans of the global marine hydrothermal ecosystems and of the oil fields located below the sea bottom, similar to hyperthermophilic members of the order *Thermococcales* [50,51]. Physicochemical conditions of the Uzen oil field also favored this group of methanogens. Methane production is probably the major geochemical process carried out by archaea of the Uzen oil field. 

In the low-sulfate formation water samples, methanogens were accompanied by anaerobic, thermophilic, and fermentative bacteria of the genera *Thermovirga*, *Defliviitoga*, and Thermosipho (order *Thermotogales*). They are characterized by the presence of a toga, an outer sheath-like structure surrounding the cells. Members of this order have been isolated from various high-temperature environments such as hot springs, oil reservoirs, and marine hydrothermal vents. Bacteria of the genera *Thermosipho* and *Defliviitoga* grow on sugars, while *Thermovirga* can utilize only proteinaceous substrates [52,53,54]. Known members of these genera are able to reduce thiosulfate or/and molecular sulfur to sulfide. 

In the sulfide-rich formation water (8533, 4256, 8001, 6928, and 9064) with different sulfate concentrations, the share of methanogens decreased significantly in favor of sulfidogenic bacteria of the genus *Desulfonauticus* (Figure 2). This genus comprises two species of thermophilic halotolerant bacteria, an organoheterotrophic *Desulfonauticus submarinus* and a chemolithoautotrophic bacterium *Desulfonauticus autotrophicus*, which were isolated from hydrotherms and from an oil field, respectively [44]. SRB of the genera *Desulfovibrio*, *Desulfomicrobium*, *Thermodesulforhabdus*, *Thermodesulfobacterium*, *Desulfotomaculum*, etc. were also present in formation water, seawater, and its mixtures with formation water (SWin, PWRI-1, PWRI-2, 2755-30 m^3^). *Desulfonauticus* sequences were detected also in high-temperature oil fields in Australia [55] and in the Alaskan Ivishak oil field (USA) [56] exploited with seawater flooding. Temperatures of the oil-bearing horizons, high salinity of formation water, and injection of seawater have also determined the distribution of the *Desulfonauticus* members in the Uzen oil field. 

Analysis of the *dsrA* gene encoding α-subunit of the dissimilatory sulfite reductase confirmed the presence of *Desulfonaticus*, *Desulfovibrio*, *Desulfomicrobium*, *Desulfotomaculum*, *Desulfoglaeba*, and *Moorella*, as well as of uncultured sulfite reducers in formation water. Among these genera, only *Desulfoglaeba* members were able to grow in media with crude oil and *n*-alkanes [57]. 

In the zone of injection well (2755-30 m^3^) and in the injection facility (PWRI-1), where the temperature (about 40–45 °C) was lower than in the oil field, methylotrophic methanogens of the genus *Methanolobus* were revealed. Members of this genus are mesophilic and have been isolated from marine sediments and from a natural gas field [58]. They probably arrived to the Uzen oil field with seawater and persisted in the injected water supply system. 

Potential nitrate-reducing bacteria of the genera *Deferribacter*, *Alcaligenes*, *Arcobacter*, *Marinobacter*, *Pseudomonas*, *Tepidiphilus*, etc. were revealed by molecular techniques in formation water from the Uzen oil field (Figures S5c, S6c, and S7c). In our experiments, even high nitrate concentrations did not result in nitrite formation and suppression of sulfidogenesis by mesophilic microorganisms from the injection zone (Figure S8). Nitrate was reduced to dinitrogen by the population of mesophilic denitrifying bacteria. In the thermophilic thiosulfate-reducing enrichments 9677 and 3239, however, sulfide formation was suppressed at 0.5–1.0 g NO_3_^−^ L^−1^, probably due to nitrite accumulation in these cultures (up to 100 mg NO_2_^−^ L^−1^) (Figure S8a,b). 

Similar results have been reported by Fida and co-workers [59] for the Terra Nova field, offshore from Newfoundland, Canada. Thermophilic microbial consortia, comprising bacteria of the genera *Petrobacter* and *Geobacillus*, reduced nitrate to nitrite at temperatures from 50 to 70 °C, while mesophilic consortia containing *Thauera* and *Pseudomonas* from the near-bottom zone of the injection well reduced nitrate and nitrite to dinitrogen at 40 and 45 °C. The authors suggested that preventing the temperature of these zones from dropping below 50 °C will limit nitrite reduction, providing for more effective souring control [59].

In laboratory experiments [60], nitrite suppressed sulfide production as long as the nitrite concentration remained above 15 mg N L^−1^. Sulfide production recovered more rapidly after nitrite treatment than after glutaraldehyde treatment. Neither glutaraldehyde nor nitrite had much effect on biofilm thickness, but both chemicals did significantly interfere with sulfide generation.

A range of *Desulfovibrio* strains are known to be resistant to high nitrate concentrations. Korte and co-workers [61] suggested that spontaneous mutations in the cultures of *Desulfovibrio vulgaris* and *Desulfovibrio alaskensis* resulted in increased nitrate resistance of some cells, which then became predominant in the population. 

The results obtained with enrichment cultures of sulfate-, thiosulfate-, and sulfur-reducing bacteria indicate the presence of various sulfidogenic prokaryotes in the oil field (Figures S2 and S9). A number of sulfate-reducing bacteria are known to use diverse electron acceptors: sulfate, sulfite, thiosulfate, and elemental sulfur [62]. Sulfide production in thiosulfate- and sulfur-reducing enrichments resulted probably mainly from the activity of fermentative bacteria carrying out transfer of reducing equivalents onto oxidized sulfur compounds. Steel corrosion, however, may occur in the absence of sulfate due to syntrophic processes carried out by different types of hydrogenotrophic microorganisms, including sulfate reducers, methanogens, and acetogens [63,64,65]. 

Bacteria of the genera *Desulfovibrio*, *Geotoga*, *Pelobacter*, *Pseudomonas*, *Acetobacterium*, and methanogenic archaea formed highly corrosive biofilms in an offshore oil production facility [66]. Several members of the genus *Pelobacter* possess the unique metabolic ability to grow both fermentatively and by reducing selenate, selenite, nitrate, poorly crystalline Fe(III), and anthraquinone disulfonate in a medium with acetate [67]. *Pelobacter* was found in oil reservoirs [68,69], as well as in the Uzen oil field. Using pure culture of *Desulfovibrio alaskensis* Kaz19, we showed that biofilms of this strain were more resistant to biocidal agents (glutaraldehyde and Rancid) than its planktonic form (Figure S10). Scanning electron microscopy of the strain Kaz19 revealed pilus-like nanowires resembling those of *Pelotomaculum thermopropionicum* [70], with which they were connected to other cells (Figure 5b). Such structures have not been previously reported for *D. alaskensis*.

Extracellular microbial nanowires (MNWs) act as a conduit of electrons between cells and distant substrates and participate in long-distance (up to micrometer) extracellular electron transfer [71]. MNWs have been observed in iron (Fe)- and sulfate-reducing bacteria, in Fe-oxidizers, and in photosynthetic bacteria. *D. alaskensis* is a bacterium capable of generating electricity in microbial fuel cells (MFCs), as was described in the review [72]. The ability of *D. alaskensis* to form biofilms and MNWs and to produce molecular hydrogen in the absence of sulfate (e.g., in the medium with formate [73,74]) make it a participant of transmembrane electron transfer and an ecologically valuable component of subterranean sulfidogenic communities.

## 5. Conclusions

While original formation water of the high-temperature Uzen oil field contained no sulfate, injection of sulfate-reach seawater resulted in activation of sulfidogenesis in the oil field, which may cause problems in oil recovery and processing. In the present work, microbial diversity in formation water and injection facility was determined by the 16S rRNA and *dsrA* gene sequencing, and the potential functional activity of microbial communities was predicted by KEGG analysis. Thermophilic halotolerant hydrogenotrophic methanogens of the genus *Methanothermococcus* exhibited the greatest functional potential in samples of formation water from the oil layer only lowly affected by injected water, while sulfate-reducing *Desulfonauticus* bacteria were most active in the zone of seawater penetration. Both mesophilic and thermophilic metabolically versatile microbial populations were revealed in the zone of injection wells with alternating oxic–anoxic conditions, where the temperature was decreased due to seawater injection. Mesophilic methylotrophic methanogens of the genus *Methanolobus*, sulfate-reducers *Desulfotomaculum*, *Desulfonauticus*, and *Desulfoglaeba*, fermentative bacteria of the genera *Bellilinea* and *Thermovirga*, potential denitrifiers *Pelobacter* and *Sulfurospirillum*, and Fe(III)-, sulfur-, and nitrate-reducers *Geoalkalibacter* were the main potential agents involved in the methane, sulfur, and nitrogen pathways. Mesophilic sulfate- and thiosulfate-reducing enrichments were resistant to nitrate concentrations of up to 2 g NO_3_^−^ L^−1^, since they included denitrifying bacteria, which reduced nitrate to dinitrogen. However, in our experiments the thermophilic denitrifying population produced nitrite from nitrate at 55 °C. Thus, nitrate addition could suppress sulfidogenesis in the layers with high temperature. These results indicate that application of nitrate even in high concentrations can hardly be recommended for suppression of sulfidogens in the near-bottom zone of injection wells where the temperature is about 40–45 °C. The data obtained show the need to use several complementary methods to suppress the growth of sulfidogens in the oil reservoir.

*D. alaskensis* strain Kaz19 formed biofilms, which allowed for its survival in the presence of biocidal agents (glutaraldehyde and Rancid). This study highlights the need to evaluate the effect of biocides on both planktonic cells and biofilms of various sulfidogenic communities present in the oil field prior to their large-scale applications. The presence of the known electricity- and biofilms-producing bacteria *Desulfovibrio*, *Pseudomonas*, *Pelobacter*, *Halomonas*, and *Marinobacter* indicates the need to clarify the effect of exoelectrogenic activity on sulfidogenesis in petroleum reservoirs with a shortage of available electron acceptors and low water and mass exchange.

## Figures and Tables

**Table 1:** Physicochemical characteristics of production and injection water samples from the Uzen oil field.

Well Number, Sample	pH	Total Salinity, mg·L^−1^	Content ^1^, mg L^−1^						H_2_S, mg L^−1^
			Ca^2+^	Mg^2+^	Na^+^ + K^+^	Cl^−^	SO_4_^2−^	HCO_3_^−^	
6928	6.7	42,534.6	2004.0	1337.6	12,417.7	26,303.9	44.4	427.0	107.6
8001	6.7	47,127.6	2204.4	1337.6	14,043.8	29,310.0	0.0	231.8	91.0
9064	6.8	30,838.9	1803.6	1094.4	8369.7	18,788.5	233.7	549.0	161.0
8533	8.0	14,039.1	1202.4	729.6	2948.6	8266.9	86.4	805.2	106.3
2874	6.5	48,765.2	2404.8	1216.0	14,618.8	30,062.0	0.0	463.6	5.6
9677	7.0	24,210.4	1202.4	972.8	6587.2	14,655.0	0.0	793.0	12.0
6916	6.2	120,556.4	6412.8	3040.0	35,737.4	75,154.0	65.8	146.4	15.6
7062	6.3	43,039.0	2004.0	1337.6	12,627.0	26,680.0	0.0	390.4	18.3
4256	6.3	80,533.4	4809.6	2432.0	22,636.6	50,399.0	0.0	256.2	7.7
6038	6.4	49,192.2	3006.0	3040.0	11,362.0	31,564.6	0.0	219.6	2.4
3257	6.2	63,139.2	4008.0	1824.0	17,631.8	39,455.8	0.0	219.6	16.2
8941	6.3	62,736.8	4408.8	1702.4	17,204.0	39,080.0	0.0	341.6	6.4
7574	6.4	36,344.6	2404.8	1702.4	8965.4	22,546.2	433.0	292.8	1.6
3239	6.6	37,902.3	2404.8	1945.6	9128.7	22,921.9	1281.7	219.6	19.9
2755-30 m^3^	6.6	51,410.5	3807.6	1459.2	13,763.2	31,941.5	48.6	390.4	2.5
PWRI-1	6.7	54,183.2	3607.2	1216.0	15,446.8	33,443.5	103.7	366.0	1.6
PWRI-2	6.6	58,570.1	4008.0	1702.4	15,991.9	36,450.0	51.8	366.0	0.0
POTP	6.6	41,362.9	3006.0	1580.8	10,511.0	25,928.1	19.8	317.2	2.4
SW inlet	7.5	12,890.8	1202.4	729.6	2267.8	5260.7	3161.9	268.4	0.0
SW outlet	7.5	11,764.2	1402.8	851.2	1444.4	4885.0	2912.4	268.4	0.5
AW	8.5	9609.5	200.4	170.2	3064.0	3907.0	2181.5	73.2	0.0
Standard error	±0.1		±0.09	±0.09	±0.02	±0.004	±0.02	±0.001	±0.02

^1^ CO_3_^2−^ was detected in the Alb-Cenomanian water sample (13.2 mg L^−1^) and was not detected in the other samples.

## Data Availability

The GenBank/EMBL/DDBJ accession number of the 16S rRNA gene sequence of *Desulfovibrio alaskensis* strain Kaz19 is MZ604343. The row data generated from 16S rRNA and *dsrAB* gene sequencing of microbial communities have been deposited in the NCBI Sequence Read Archive (SRA) under the accession numbers SRR15234354–SRR15234380 (Bioproject PRJNA749317) and SRR15293245, SRR15293278–SRR15293283 (Bioproject PRJNA749166), respectively.

## Supplementary Materials

The Supplementary Material for this article can be found online at https://www.mdpi.com/article/10.3390/microorganisms9091818/s1. Table S1: Number of sequences and diversity indices in the libraries of the 16S rRNA gene fragments from the microorganisms of injection and production water samples from the Uzen oil field, Table S2: Diversity indices in the libraries of the bacterial 16S rRNA gene fragments from sulfate-reducing (SR), thiosulfate-reducing (TSR), and denitrifying (DNB) enrichments obtained from water samples collected from the near-bottom zone of an injection well 2755 (30 m^3^) and from petroleum oil treatment plant (POTP) of the Uzen oil field, Figure S1: Numbers of aerobic organotrophic bacteria (AOB) and anaerobic fermentative (Ferm), sulfate-reducing (SRP) and methanogenic (MG) prokaryotes in injection and production water samples from the Uzen oil field. Microorganisms from Kaspian Sea water (SWin and SWout) were incubated at 25 °C, from PWRI-1, PWRI-2, and POTP—at 42 °C, from production wells and of Alb-Cenomanian water—at 55 °C, Figure S2: Sulfide production in sulfate-reducing (SO_4_^2−^-red.), sulfur-reducing (S^0^-red.), and thiosulfate-reducing (S_2_O_3_^2−^-red.) enrichments obtained from the Uzen oil field, Figure S3: Taxonomic classification of 16S rRNA gene fragments in the libraries from water samples from the Uzen oil field at the Bacteria and Archaea domain level using the RDP classifier, Figure S4: Taxonomic classification of dsrA gene fragments in the libraries from injection and production water samples at the phylum (a) and genus (b) level using the RDP classifier. The taxa constituting at least >1% in each library are listed, Figure S5: Heatmaps highlighting the key contributing microorganisms involved in the “Methane metabolism” (a), “Sulfur metabolism” (b), and “Nitrogen metabolism”(c) pathways in samples of injection and production water from the Uzen oil field, Figure S6: Predicted enzyme profile for “Methane metabolism” (a), “Sulfur metabolism” (b), and “Nitrogen metabolism” (c) pathways of microorganisms in the sample from the near-bottom zone of injection well 2755-30. The graphs were generated with KEGG Color mapper tool (http://www.genome.jp/kegg/tool/map_pathway3.html accessed on 15 July 2021) using the Color Mapping files generated by Local Mapper module of iVikodak. Color intensity indicates an average gene (enzyme) proportion in sample. The pathways probably employed by microorganisms in the sample are marked by red, Figure S7: Predicted enzyme profile for “Methane metabolism” (a), “Sulfur metabolism” (b), and “Nitrogen metabolism” (c) pathways of microorganisms in the water sample from production well 7062. The graphs were generated with KEGG Color mapper tool (http://www.genome.jp/kegg/tool/map_pathway3.html accessed on 15 July 2021) using the Color Mapping files generated by Local Mapper module of iVikodak. Color intensity indicates an average gene (enzyme) proportion in sample. The pathways probably employed by microorganisms in the sample are marked by red, Figure S8: Production of sulfide (a) and nitrite (b) by thiosulfate-reducing (TSR) and sulfate-reducing (SR) bacteria of injection and production water samples in the media with different initial nitrate concentrations. Enrichments 2755-30, PWRI-1, PWRI-2, and POTP were incubated at 42 °C; enrichments 9677, 3239, AW, and 6038 were incubated at 55 °C Cultivation duration was 30 days, Figure S9: The numbers of sequences in the libraries and bacterial diversity in sulfate-reducing (SR), thiosulfate-reducing (TSR), and denitrifying (DNB) enrichments obtained from the near-bottom zone of injection well 2755 (30 m^3^) (A) and from petroleum oil treatment plant (POTP) (B) at the phylum (a, d, g), family (b, e, h), and genus (c, f, i) levels based on the 16S rRNA sequence analysis. The total numbers of sequences in libraries of SR, TSR and DNB enrichments from 2755-30 sample are 88743, 83263, and 82409, and from POTP sample are 59710, 73376, and 90139, respectively, Figure S10: Effect of glutaraldehyde and Rancid on sulfide production (a) and survival (b) of sulfate-reducing bacterium D. alaskensis strain Kaz19 in planktonic form and biofilms. Designations in figure (a): Control medium without biocides (Line 1) and with Rancid at concentration 40 (Line 2), 80 (Line 3), and 120 mg·L^−1^ (Line 4) and with glutaraldehyde at concentrations 100 (Line 5), 250 (Line 6), and 500 mg·L^−1^ (Line 7).
