# Supplementary material for: Sulfidogenic Microbial Communities of the Uzen High-Temperature Oil Field in Kazakhstan
Source: Microorganisms. 2021 Aug 26;9(9):1818. doi: 10.3390/microorganisms9091818 (PMC8467725; doi:10.3390/microorganisms9091818)
Supplement: Supplementary file 1 [file microorganisms-09-01818-s001.zip › Supplementary Material-rev3.pdf]

Supplementary Materials

Sulfidogenic microbial communities of the Uzen high-temperature oil field in Kazakhstan

Diyana S. Sokolova [1], Ekaterina M. Semenova [1], Denis S. Grouzdev [2], Salimat K. Bidzhieva [1], Tamara L. Babich [1], Nataliya G. Loiko [1], Alexey P. Ershov [1], Vitaliy V. Kadnikov [4], Alexey V. Beletsky [4], Andrey V. Mardanov [4], Nurlan S. Zhaparov [5] and Tamara N. Nazina [1,*]

[1] Winogradsky Institute of Microbiology, Research Center of Biotechnology of the Russian Academy of Sciences, Moscow 119071, Russia; sokolovadiyana@gmail.com (D.S.S.); semenova_inmi@mail.ru (E.M.S.); salima.bidjieva@gmail.com (S.K.B.); microb101@yandex.ru (T.L.B.); loikonat@mail.ru (N.G.L.); e.alexey.mail@yandex.ru (A.P.E.); nazina@inmi.ru (T.N.N.)

[2] SciBear OU, Tallinn, Estonia; denisgrouzdev@gmail.com (D.S.G.)

[3] Institute of Bioengineering, Research Center of Biotechnology of the Russian Academy of Sciences, Moscow, 119071, Russia; vkadnikov@bk.ru (V.V.K.); mortu@yandex.ru (A.V.B.); mardanov@biengi.ac.ru (A.V.M.)

[4] Branch of the Limited Liability Partnership "KazMunaiGas Engineering", Aktau, Kazakstan; zhaparov_n@kaznipi.kz (N.S.Z.)

* Correspondence: nazina@inmi.ru; Tel.: +7-499-135-0341 (T.N.N.)

Table S1. Number of sequences and diversity indices in the libraries of the 16S rRNA gene fragments from the microorganisms of injection and production water samples from the Uzen oil field.

Parameters	6928	8001	9064	8533	PWRI-1	SWin	SWout	PWRI-2	2874	9677	6916
Number of reads	64500	148657	52623	53443	36839	116602	91268	65611	32435	85	76110
OTUs number	245	139	172	655	607	306	119	579	223	34	174
Shannon index (H10)	2.5	1.97	2.37	0.42	0.39	0.33	0.46	0.36	0.69	0.45	1.31
Simpson index (1-D)	0.447	0.507	0.462	0.896	0.908	0.939	0.887	0.925	0.774	0.897	0.648
	**7062**	**POTP**	**6038**	**3257**	**8941**	**7574**	**3239**	**AW**	**4256**	**2755-30**	
Number of reads	81528	26490	99504	69373	118765	72817	78836	84297	84025	28285	
OTUs number	119	363	142	123	186	230	316	75	240	522	
Shannon index (H10)	2.24	0.56	4.05	3.37	1.23	0.7	0.8	1.34	0.49	0.55	
Simpson index (1-D)	0.455	0.827	0.339	0.367	0.655	0.767	0.736	0.606	0.871	0.84	

Table S2. Diversity indices in the libraries of the bacterial 16S rRNA gene fragments from sulfate-reducing (SR), thiosulfate-reducing (TSR), and denitrifying (DNB) enrichments obtained from water samples collected from the near-bottom zone of an injection well 2755 (30 m^3) and from petroleum oil treatment plant (POTP) of the Uzen oil field.

Sample name	Target reads	OTUs	ACE	CHAO	Shannon	Simpson	Phylogenetic diversity	Good's coverage of library (%)
SR-2755-30	88743	97	100.08	97.81	0.95	0.59	125	99.99
TSR-2755-30	83263	322	332.85	325.61	2.24	0.20	299	99.97
DNB-2755-30	82409	244	254.20	246.92	2.65	0.12	264	99.98
SR-POTP	59710	278	285.92	283.00	1.72	0.39	537	99.97
TSR-POTP	73376	216	222.33	217.44	1.40	0.44	209	99.98
DNB-POTP	90139	72	87.34	80.50	1.10	0.55	176	99.98

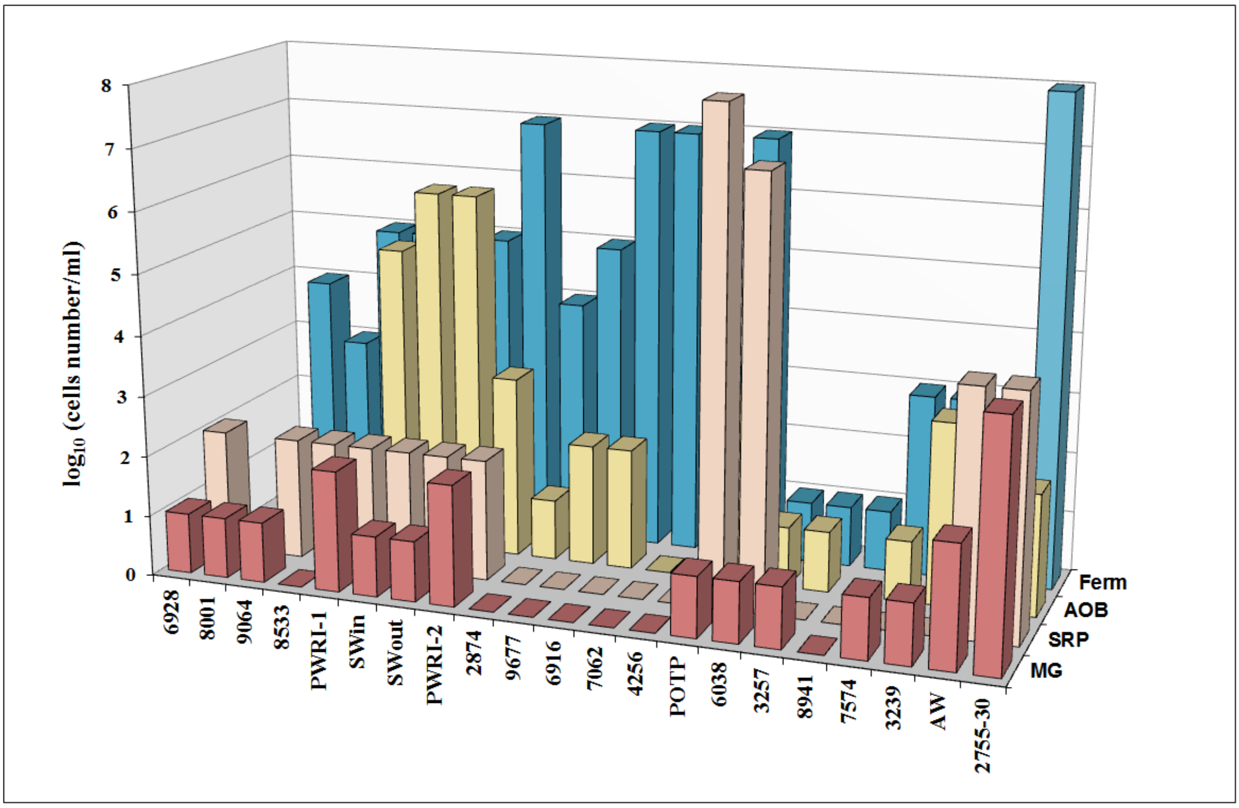

Figure S1. Numbers of aerobic organotrophic bacteria (AOB) and anaerobic fermentative (Ferm), sulfate-reducing (SRP) and methanogenic (MG) prokaryotes in injection and production water samples from the Uzen oil field. Microorganisms from Kaspian Sea water (SWin and SWout) were incubated at 25 °C, from PWRI-1, PWRI-2, and POTP – at 42 °C, from production wells and of Alb-Cenomanian water – at 55 °C.

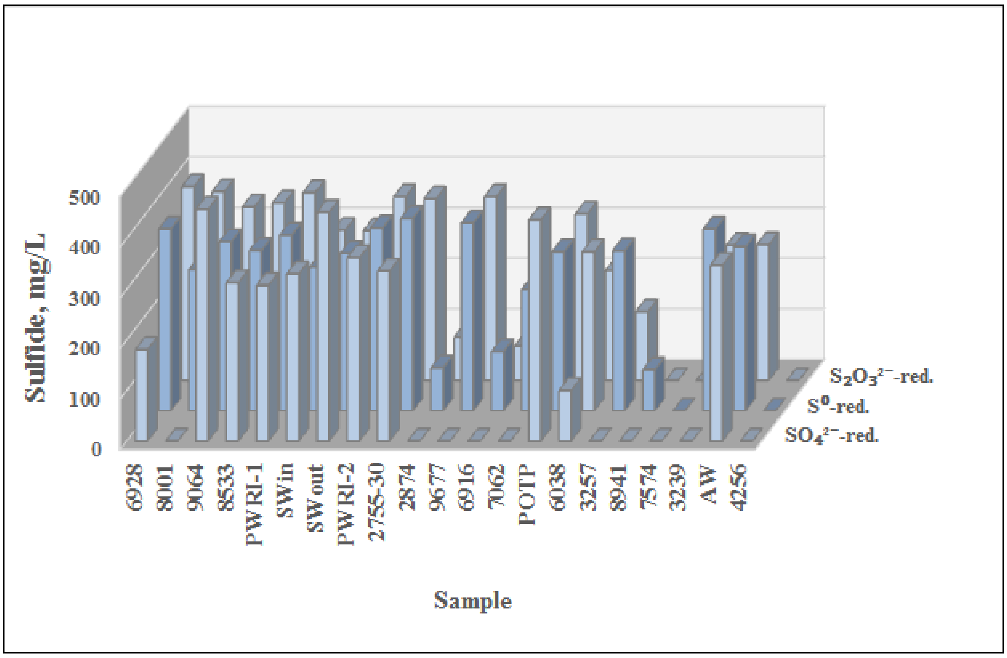

Figure S2. Sulfide production in sulfate-reducing (SO_4^{2-}-red.), sulfur-reducing (S^0-red.), and thiosulfate-reducing ($S_2O_3^{2-}$-red.) enrichments obtained from the Uzen oil field.

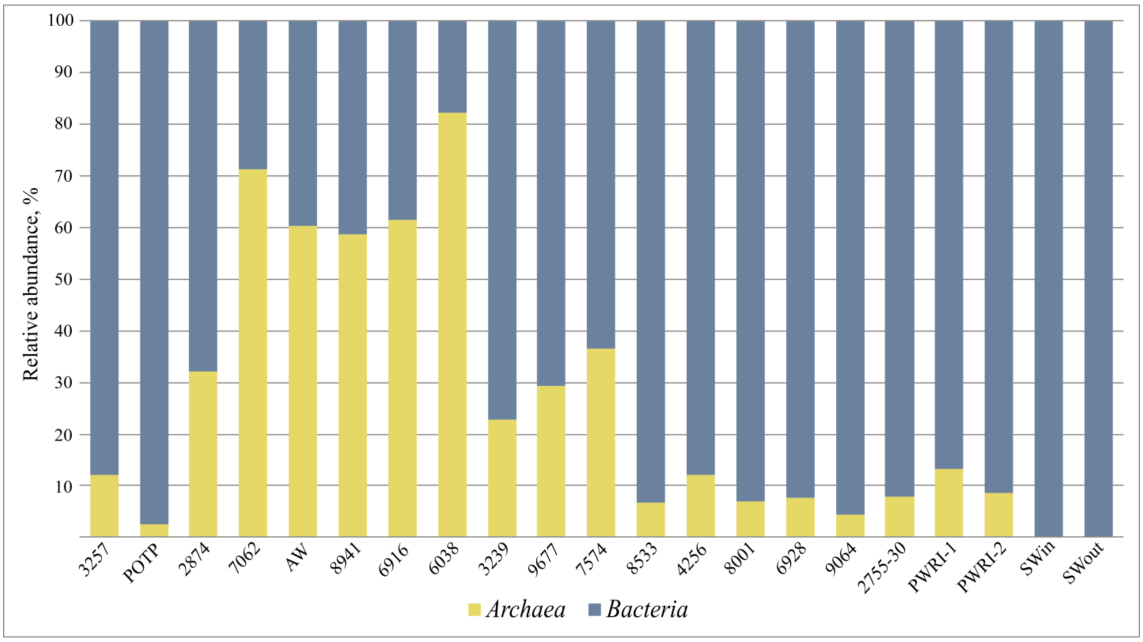

Figure S3. Taxonomic classification of 16S rRNA gene fragments in the libraries from water samples from the Uzen oil field at the *Bacteria* and *Archaea* domain level using the RDP classifier.

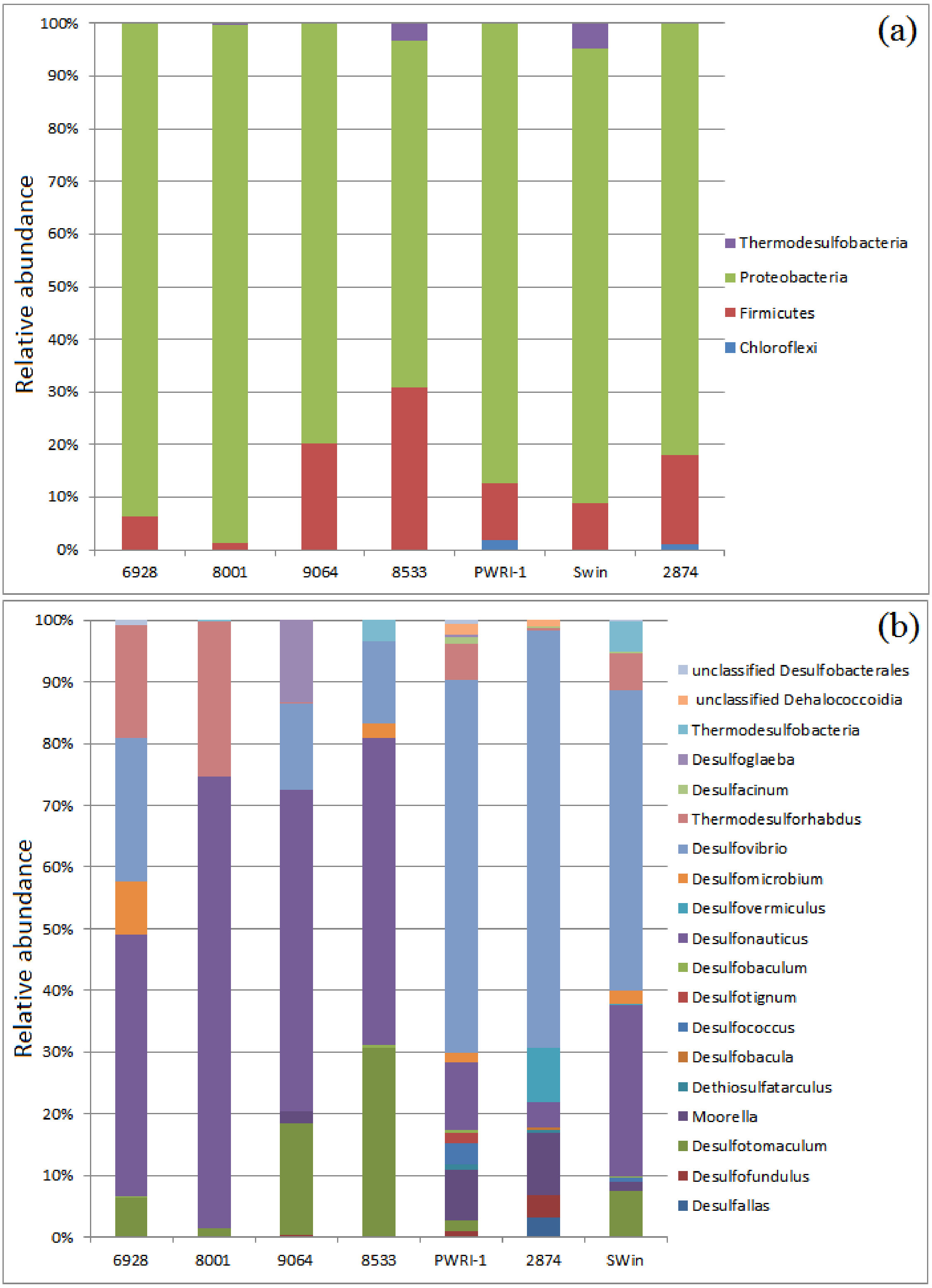

Figure S4. Taxonomic classification of *dsrA* gene fragments in the libraries from injection and production water samples at the phylum (a) and genus (b) level using the RDP classifier. The taxa constituting at least >1% in each library are listed.

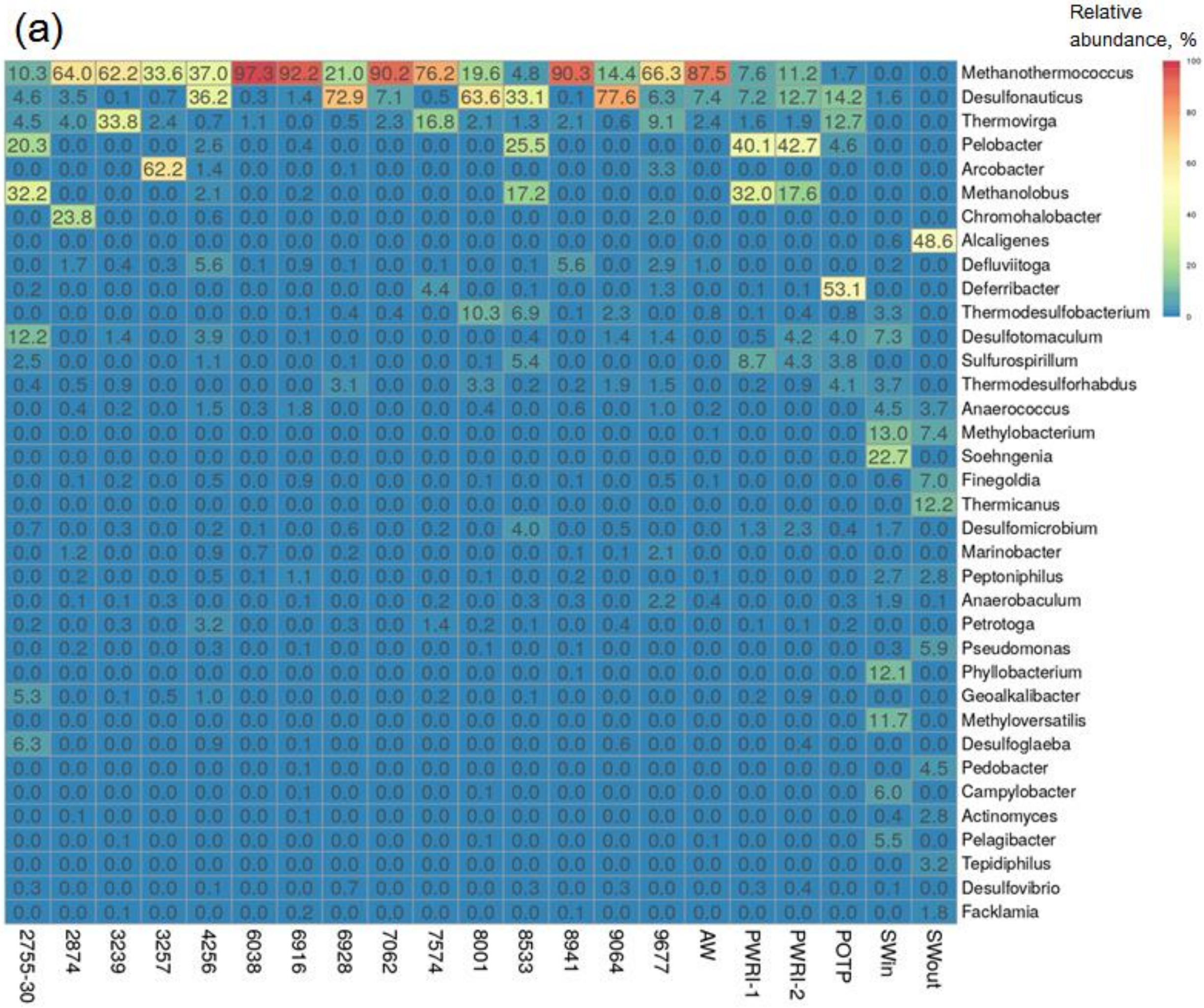
(a)
Relative abundance, %
Methanothermococcus
Desulfonauticus
Thermovirga
Pelobacter
Arcobacter
Methanolobus
Chromohalobacter
Alcaligenes
Defluviitoga
Deferribacter
Thermodesulfobacterium
Desulfotomaculum
Sulfurospirillum
Thermodesulforhabdus
Anaerococcus
Methylobacterium
Soehngenia
Finegoldia
Thermicanus
Desulfomicrobium
Marinobacter
Peptoniphilus
Anaerobaculum
Petrotoga
Pseudomonas
Phyllobacterium
Geoalkalibacter
Methyloversatilis
Desulfoglaeba
Pedobacter
Campylobacter
Actinomyces
Pelagibacter
Tepidiphilus
Desulfovibrio
Facklamia
2755-30
2874
3239
3257
4256
6038
6916
6928
7062
7574
8001
8533
8941
9064
9677
AW
PWRI-1
PWRI-2
POTP
SWin
SWout

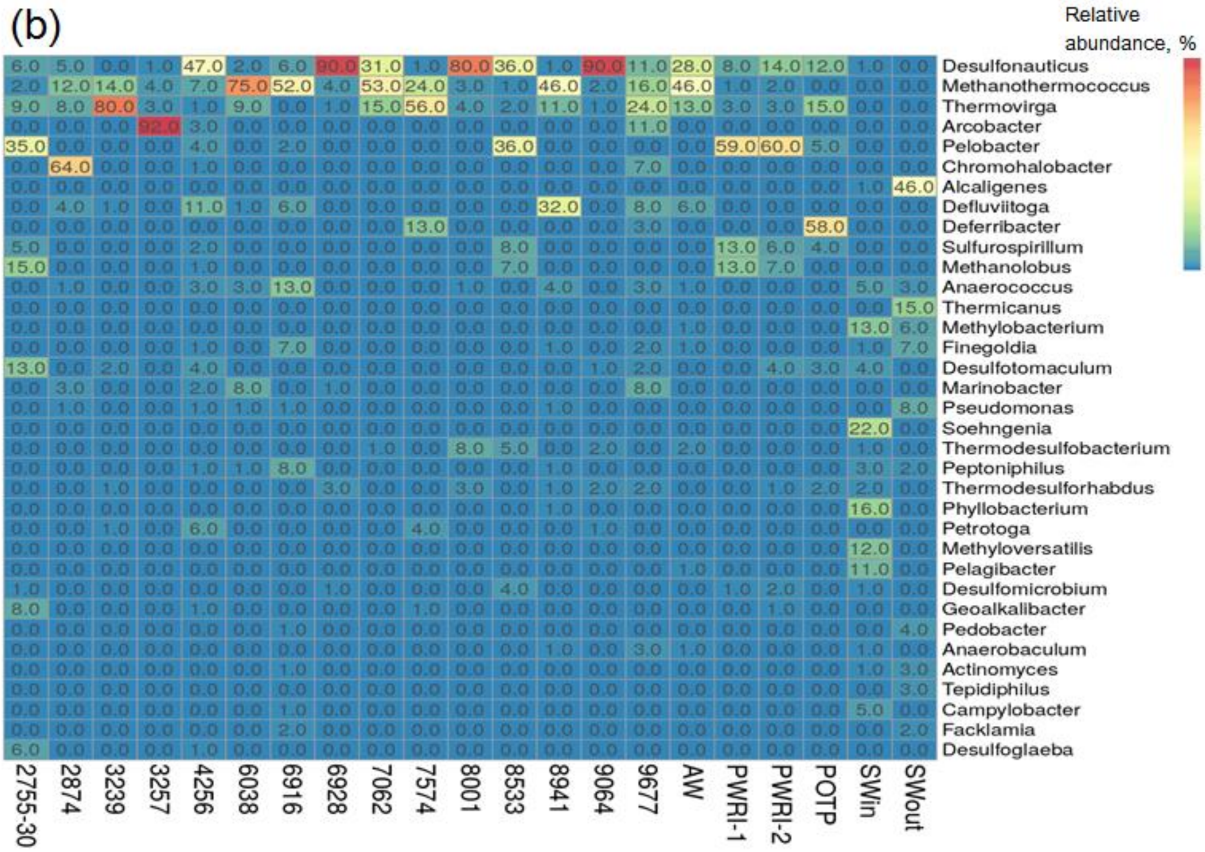
(b)
Relative abundance, %
Desulfonauticus
Methanothermococcus
Thermovirga
Arcobacter
Pelobacter
Chromohalobacter
Alcaligenes
Defluviitoga
Deferribacter
Sulfurospirillum
Methanolobus
Anaerococcus
Thermicanus
Methylobacterium
Finegoldia
Desulfotomaculum
Marinobacter
Pseudomonas
Soehngenia
Thermodesulfobacterium
Peptoniphilus
Thermodesulforhabdus
Phyllobacterium
Petrotoga
Methyloversatilis
Pelagibacter
Desulfomicrobium
Geoalkalibacter
Pedobacter
Anaerobaculum
Actinomyces
Tepidiphilus
Campylobacter
Facklamia
Desulfoglaeba
2755-30
2874
3239
3257
4256
6038
6916
6928
7062
7574
8001
8533
8941
9064
9677
AW
PWRI-1
PWRI-2
POTP
SWin
SWout

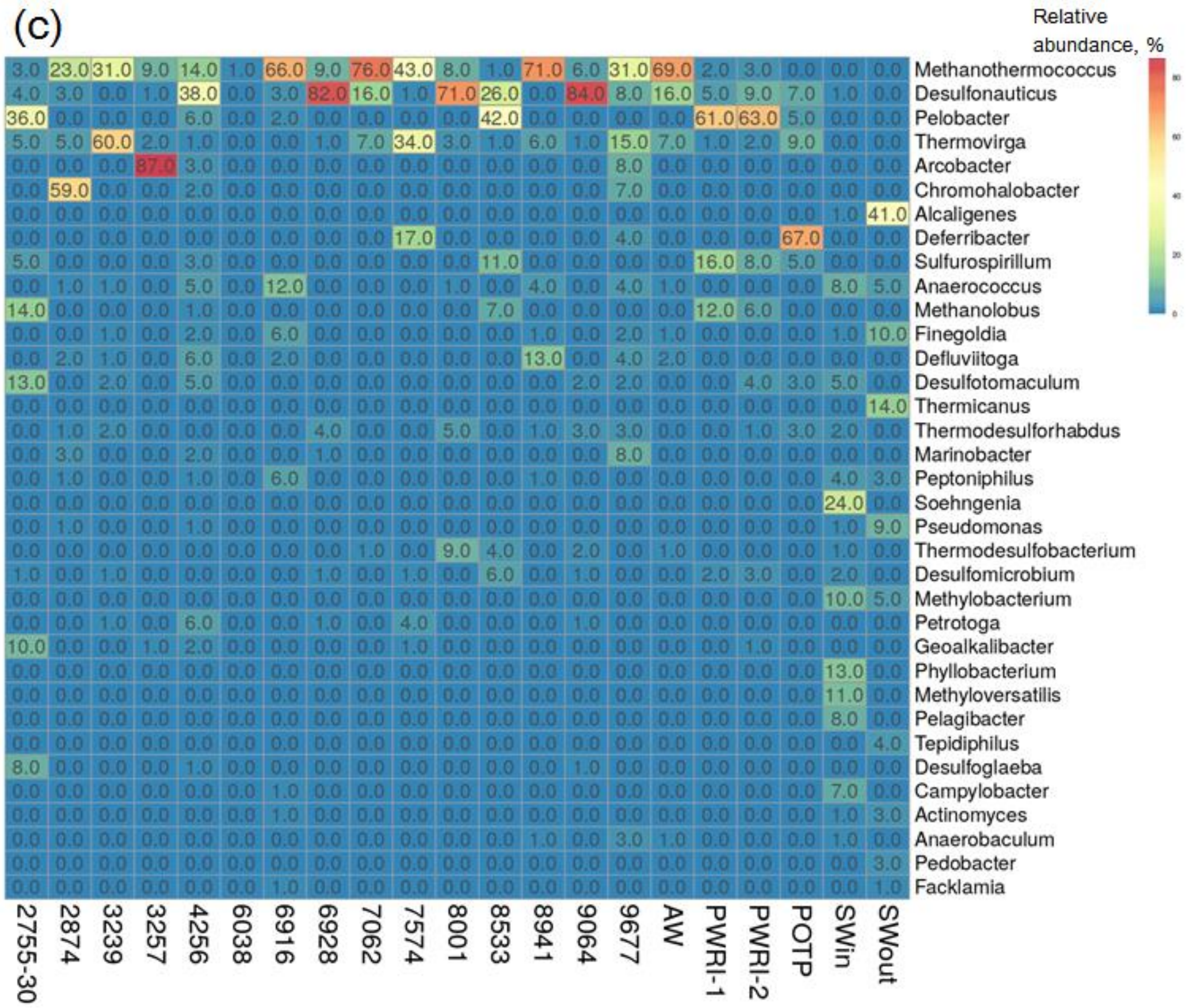

Figure S5. Heatmaps highlighting the key contributing microorganisms involved in the "Methane metabolism" (a), "Sulfur metabolism" (b), and "Nitrogen metabolism"(c) pathways in samples of injection and production water from the Uzen oil field.

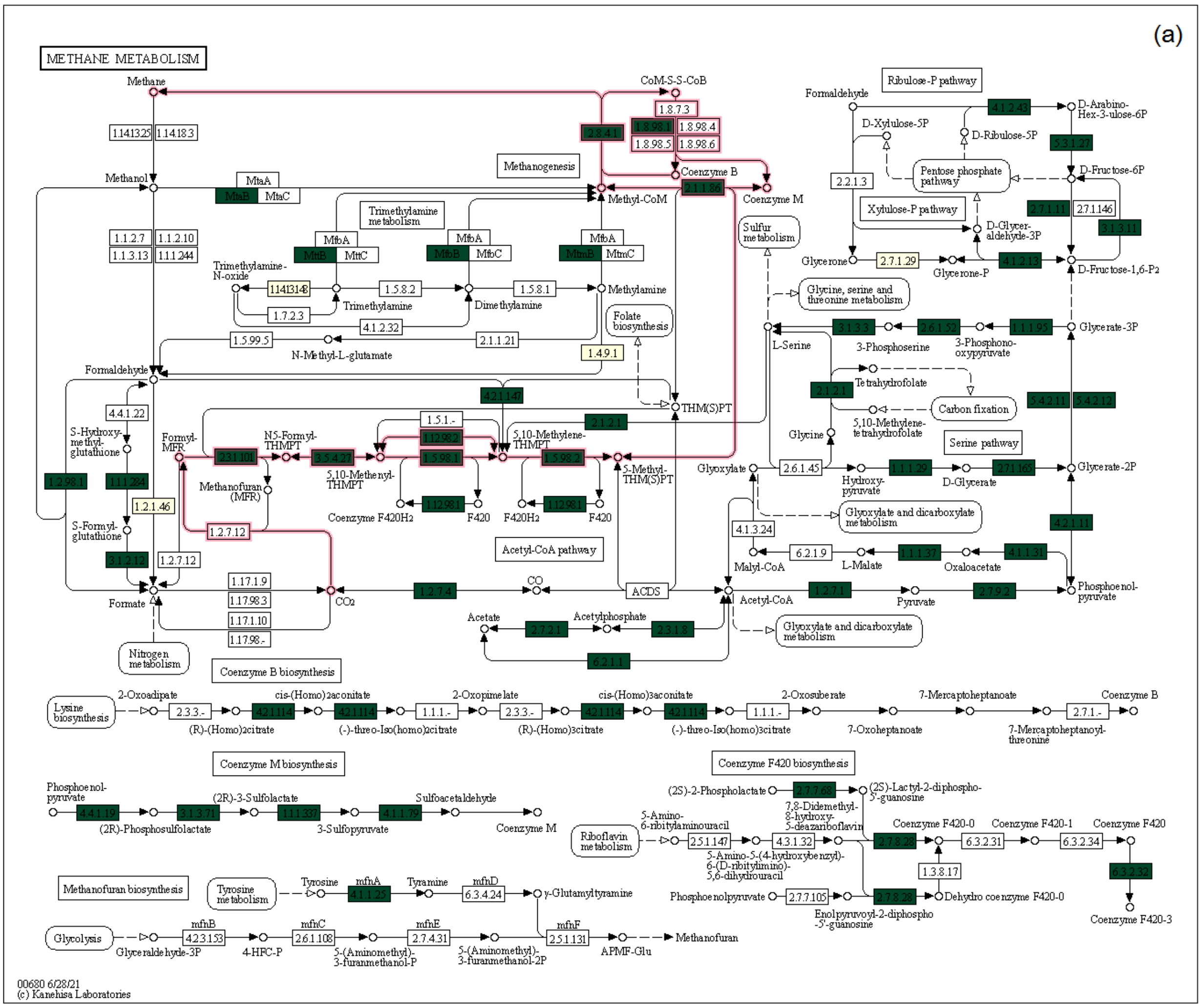
(a)
METHANE METABOLISM
Methane
CoM-S-S-CoB
Methanol
Methyl-CoM
Coenzyme B
Coenzyme M
Methanogenesis
Trimethylamine metabolism
Trimethylamine-N-oxide
Trimethylamine
Dimethylamine
Methylamine
N-Methyl-L-glutamate
Formaldehyde
Folate biosynthesis
Sulfur metabolism
S-Hydroxy-methyl-glutathione
S-Formyl-glutathione
Formate
Formyl-MFR
N5-Formyl-THMPT
5,10-Methenyl-THMPT
5,10-Methylene-THMPT
5-Methyl-THM(S)PT
THM(S)PT
Methanofuran (MFR)
Coenzyme F420H2
F420
F420H2
CO2
CO
ACDS
Acetyl-CoA pathway
Acetate
Acetylphosphate
Acetyl-CoA
Nitrogen metabolism
Ribulose-P pathway
Formaldehyde
D-Arabino-Hex-3-ulose-6P
D-Xylulose-5P
D-Ribulose-5P
D-Fructose-6P
Pentose phosphate pathway
Xylulose-P pathway
D-Glyceraldehyde-3P
Glycerone
Glycerone-P
D-Fructose-1,6-P2
Glycine, serine and threonine metabolism
L-Serine
3-Phosphoserine
3-Phosphonooxypyruvate
Glycerate-3P
Tetrahydrofolate
Carbon fixation
5,10-Methylene-tetrahydrofolate
Glycine
Serine pathway
Glyoxylate
Hydroxypyruvate
D-Glycerate
Glycerate-2P
Glyoxylate and dicarboxylate metabolism
Malyl-CoA
L-Malate
Oxaloacetate
Phosphoenolpyruvate
Pyruvate
Coenzyme B biosynthesis
Lysine biosynthesis
2-Oxoadipate
(R)-(Homo)2citrate
cis-(Homo)2aconitate
(-)-threo-Iso(homo)2citrate
2-Oxopimelate
(R)-(Homo)3citrate
cis-(Homo)3aconitate
(-)-threo-Iso(homo)3citrate
2-Oxosuberate
7-Oxoheptanoate
7-Mercaptoheptanoate
7-Mercaptoheptanoyl-threonine
Coenzyme B
Coenzyme M biosynthesis
Phosphoenol-pyruvate
(2R)-Phosphosulfolactate
(2R)-3-Sulfolactate
3-Sulfopyruvate
Sulfoacetaldehyde
Coenzyme M
Coenzyme F420 biosynthesis
(2S)-2-Phospholactate
(2S)-Lactyl-2-diphospho-5'-guanosine
5-Amino-6-ribitylaminouracil
7,8-Didemethyl-8-hydroxy-5-deazariboflavin
5-Amino-5-(4-hydroxybenzyl)-6-(D-ribitylimino)-5,6-dihydrouracil
Riboflavin metabolism
Coenzyme F420-0
Coenzyme F420-1
Coenzyme F420
Phosphoenolpyruvate
Enolpyruvoyl-2-diphospho-5'-guanosine
Dehydro coenzyme F420-0
Coenzyme F420-3
Methanofuran biosynthesis
Tyrosine metabolism
Tyrosine
Tyramine
γ-Glutamyltyramine
Glycolysis
Glyceraldehyde-3P
4-HFC-P
5-(Aminomethyl)-3-furanmethanol-P
5-(Aminomethyl)-3-furanmethanol-2P
APMF-Glu
Methanofuran
mfnA
mfnB
mfnC
mfnD
mfnE
mfnF
MtaA
MtaC
MtbA
MttC
MtbC
MtmC
00680 6/28/21
(c) Kanehisa Laboratories

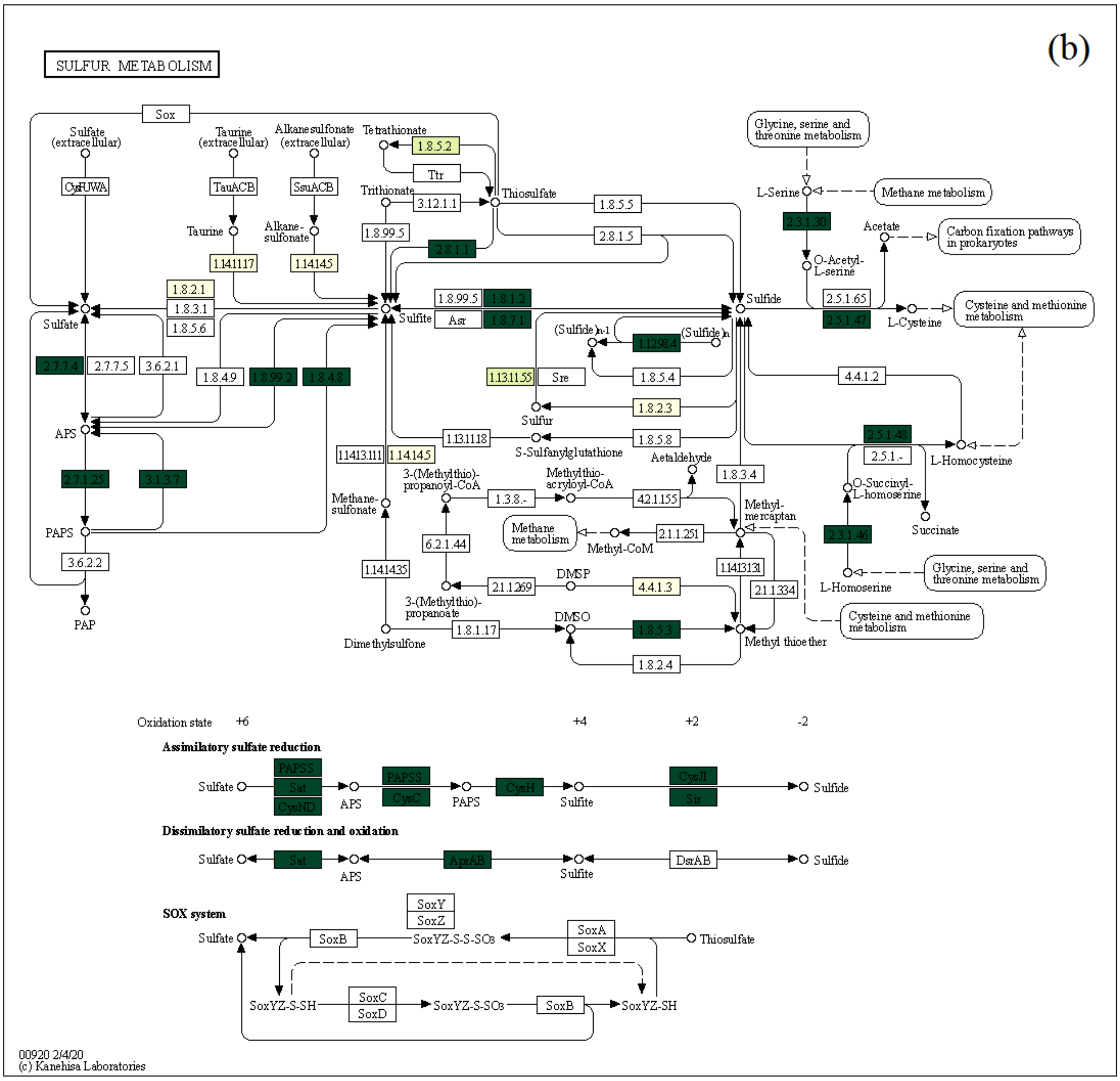
(b)
SULFUR METABOLISM
Sox
Sulfate (extracellular)
Taurine (extracellular)
Alkanesulfonate (extracellular)
Tetrathionate
1.8.5.2
Ttr
Trithionate
3.12.1.1
Thiosulfate
1.8.5.5
2.8.1.5
CysUWA
TauACB
SsuACB
Taurine
Alkane-sulfonate
1.8.99.5
2.8.1.1
1.14.11.17
1.14.14.5
1.8.2.1
1.8.3.1
1.8.5.6
Sulfate
Sulfite
1.8.99.5
1.8.1.2
Asr
1.8.7.1
Sulfide
(Sulfide)n-1
(Sulfide)n
1.12.98.4
2.7.7.4
2.7.7.5
3.6.2.1
1.8.4.9
1.8.99.2
1.8.4.8
1.13.11.55
Sre
1.8.5.4
1.8.2.3
Sulfur
APS
1.13.11.18
1.8.5.8
S-Sulfanylglutathione
1.14.13.111
1.14.14.5
2.7.1.25
3.1.3.7
Methane-sulfonate
3-(Methylthio)-propanoyl-CoA
1.3.8.-
Methylthio-acryloyl-CoA
4.2.1.155
Aetaldehyde
1.8.3.4
Methyl-mercaptan
PAPS
6.2.1.44
Methane metabolism
Methyl-CoM
2.1.1.251
1.14.14.35
1.14.13.131
3.6.2.2
3-(Methylthio)-propanoate
2.1.1.269
DMSP
4.4.1.3
2.1.1.334
PAP
Dimethylsulfone
1.8.1.17
DMSO
1.8.5.3
Methyl thioether
1.8.2.4
Glycine, serine and threonine metabolism
L-Serine
Methane metabolism
2.3.1.30
Acetate
Carbon fixation pathways in prokaryotes
O-Acetyl-L-serine
2.5.1.65
2.5.1.47
L-Cysteine
Cysteine and methionine metabolism
4.4.1.2
2.5.1.48
2.5.1.-
L-Homocysteine
O-Succinyl-L-homoserine
Succinate
2.3.1.46
L-Homoserine
Glycine, serine and threonine metabolism
Cysteine and methionine metabolism
Oxidation state
+6
+4
+2
-2
Assimilatory sulfate reduction
Sulfate
PAPSS
Sat
CysND
APS
PAPSS
CysC
PAPS
CysH
Sulfite
CysJI
Sir
Sulfide
Dissimilatory sulfate reduction and oxidation
Sulfate
Sat
APS
AprAB
Sulfite
DsrAB
Sulfide
SOX system
Sulfate
SoxB
SoxY
SoxZ
SoxYZ-S-S-SO3
SoxA
SoxX
Thiosulfate
SoxYZ-S-SH
SoxC
SoxD
SoxYZ-S-SO3
SoxB
SoxYZ-SH
00920 2/4/20
(c) Kanehisa Laboratories

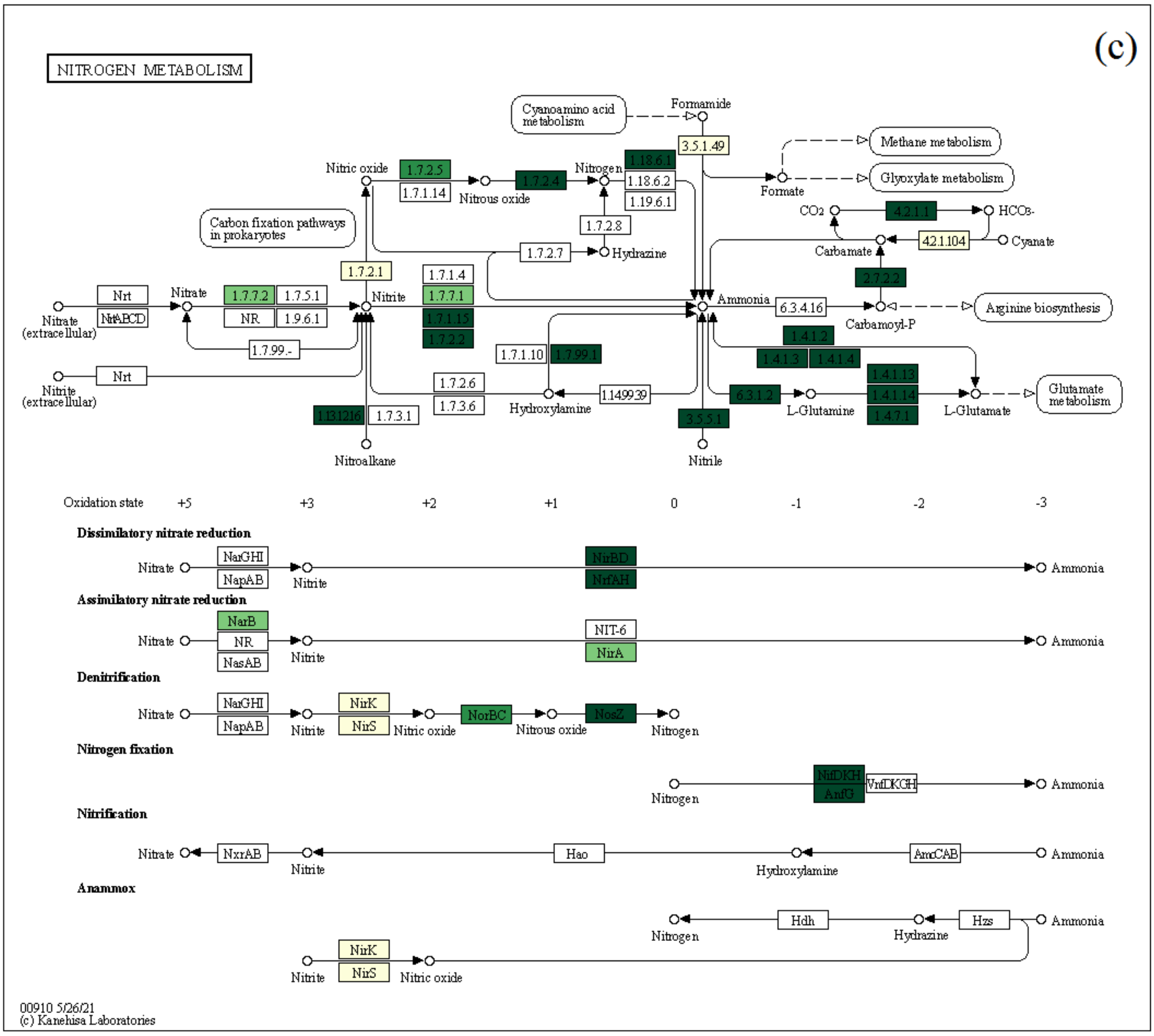

Figure S6. Predicted enzyme profile for "Methane metabolism" (a), "Sulfur metabolism" (b), and "Nitrogen metabolism" (c) pathways of microorganisms in the sample from the near-bottom zone of injection well 2755-30. The graphs were generated with KEGG Color mapper tool (http://www.genome.jp/kegg/tool/map_pathway3.html) using the Color Mapping files generated by Local Mapper module of iVikodak. Color intensity indicates an average gene (enzyme) proportion in sample. The pathways probably employed by microorganisms in the sample are marked by red.

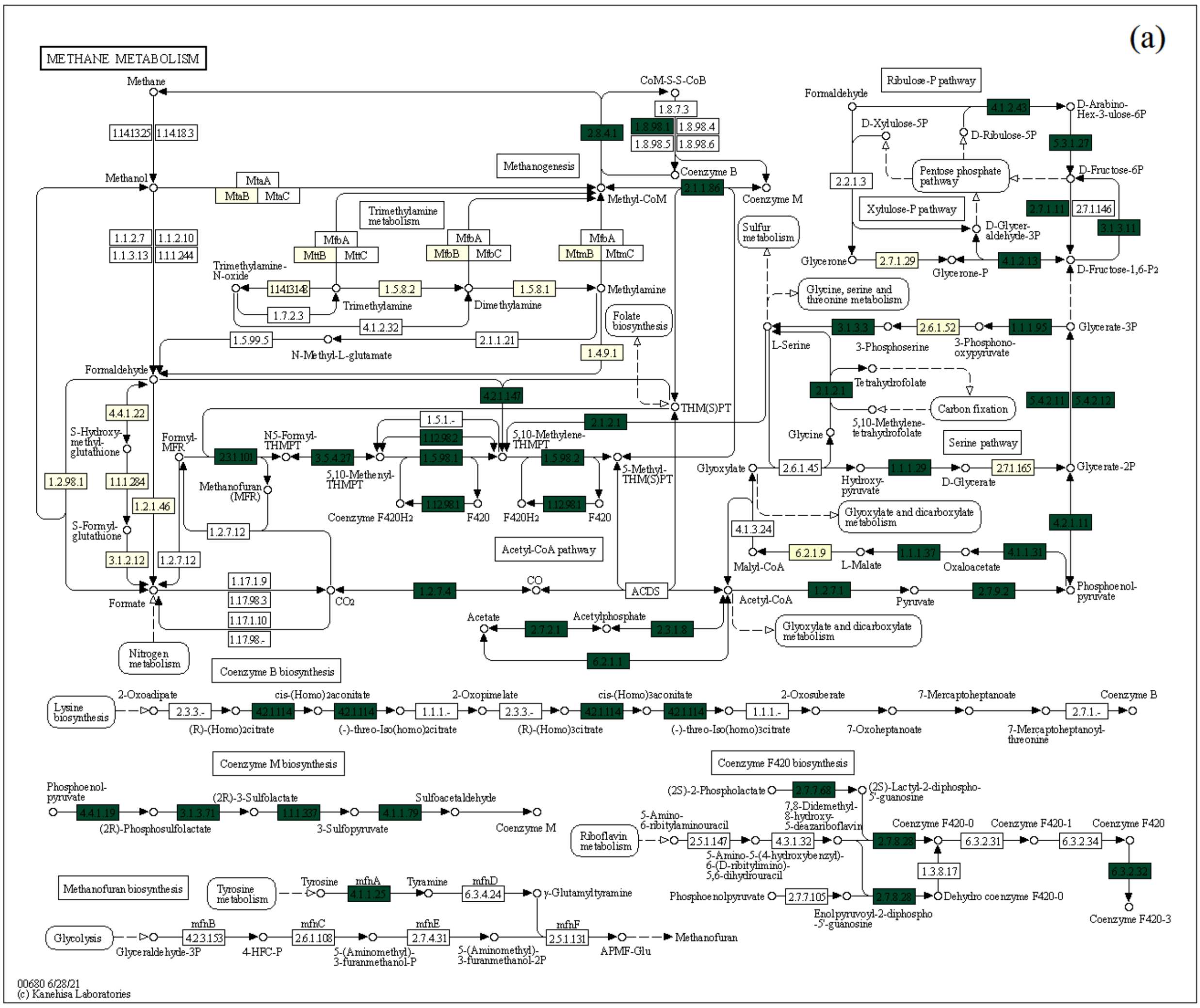
(a)
METHANE METABOLISM
Methane
CoM-S-S-CoB
1.8.7.3
1.8.98.4
1.8.98.5
1.8.98.6
1.14.18.3
Methanol
MtaA
MtaB
MtaC
Methanogenesis
Coenzyme B
Methyl-CoM
Coenzyme M
Trimethylamine metabolism
1.1.2.7
1.1.2.10
1.1.3.13
1.1.2.44
MttA
MttB
MttC
MtbA
MtbB
MtbC
MtmA
MtmB
MtmC
Trimethylamine-N-oxide
1.5.8.2
1.5.8.1
Trimethylamine
Dimethylamine
Methylamine
1.7.2.3
4.1.2.32
1.5.99.5
2.1.1.21
N-Methyl-L-glutamate
Folate biosynthesis
1.4.9.1
Formaldehyde
4.4.1.22
S-Hydroxy-methyl-glutathione
1.2.98.1
1.1.1.284
1.2.1.46
S-Formyl-glutathione
3.1.2.12
1.2.7.12
Formyl-MFR
N5-Formyl-THMPT
5,10-Methenyl-THMPT
1.5.1.-
5,10-Methylene-THMPT
5-Methyl-THMPT
THM(S)PT
Methanofuran (MFR)
Coenzyme F420H2
F420
F420H2
Acetyl-CoA pathway
1.17.1.9
1.17.98.3
1.17.1.10
1.17.98.-
Formate
CO2
CO
ACDS
Acetyl-CoA
Acetate
Acetylphosphate
Nitrogen metabolism
Coenzyme B biosynthesis
Ribulose-P pathway
D-Arabino-Hex-3-ulose-6P
D-Xylulose-5P
D-Ribulose-5P
2.2.1.3
Pentose phosphate pathway
D-Fructose-6P
Xylulose-P pathway
2.7.1.146
D-Glyceraldehyde-3P
Glycerone
2.7.1.29
Glycerone-P
D-Fructose-1,6-P2
Sulfur metabolism
Glycine, serine and threonine metabolism
L-Serine
3-Phosphoserine
2.6.1.52
3-Phosphonooxypyruvate
Glycerate-3P
Tetrahydrofolate
Carbon fixation
Glycine
5,10-Methylene-tetrahydrofolate
Serine pathway
Glyoxylate
2.6.1.45
Hydroxy-pyruvate
D-Glycerate
2.7.1.165
Glycerate-2P
Glyoxylate and dicarboxylate metabolism
4.1.3.24
Malyl-CoA
6.2.1.9
L-Malate
Oxaloacetate
Phosphoenol-pyruvate
Pyruvate
Lysine biosynthesis
2-Oxoadipate
2.3.3.-
(R)-(Homo)2citrate
cis-(Homo)2aconitate
(-)-threo-Iso(homo)2citrate
1.1.1.-
2-Oxopimelate
(R)-(Homo)3citrate
cis-(Homo)3aconitate
(-)-threo-Iso(homo)3citrate
2-Oxosuberate
7-Oxoheptanoate
7-Mercaptoheptanoate
7-Mercaptoheptanoyl-threonine
2.7.1.-
Coenzyme M biosynthesis
Coenzyme F420 biosynthesis
(2S)-2-Phospholactate
(2S)-Lactyl-2-diphospho-5'-guanosine
(2R)-3-Sulfolactate
(2R)-Phosphosulfolactate
3-Sulfopyruvate
Sulfoacetaldehyde
Riboflavin metabolism
5-Amino-6-ribitylaminouracil
2.5.1.147
4.3.1.32
7,8-Didemethyl-8-hydroxy-5-deazariboflavin
5-Amino-5-(4-hydroxybenzyl)-6-(D-ribitylimino)-5,6-dihydrouracil
Coenzyme F420-0
Coenzyme F420-1
6.3.2.31
6.3.2.34
1.3.8.17
Methanofuran biosynthesis
Tyrosine metabolism
Tyrosine
mfnA
Tyramine
mfnD
6.3.4.24
γ-Glutamyltyramine
Phosphoenolpyruvate
2.7.7.105
Enolpyruvoyl-2-diphospho-5'-guanosine
Dehydro coenzyme F420-0
Coenzyme F420-3
Glycolysis
mfnB
4.2.3.153
Glyceraldehyde-3P
4-HFC-P
mfnC
2.6.1.108
5-(Aminomethyl)-3-furanmethanol-P
mfnE
2.7.4.31
5-(Aminomethyl)-3-furanmethanol-2P
mfnF
2.5.1.131
APMF-Glu
Methanofuran
00680 6/28/21
(c) Kanehisa Laboratories

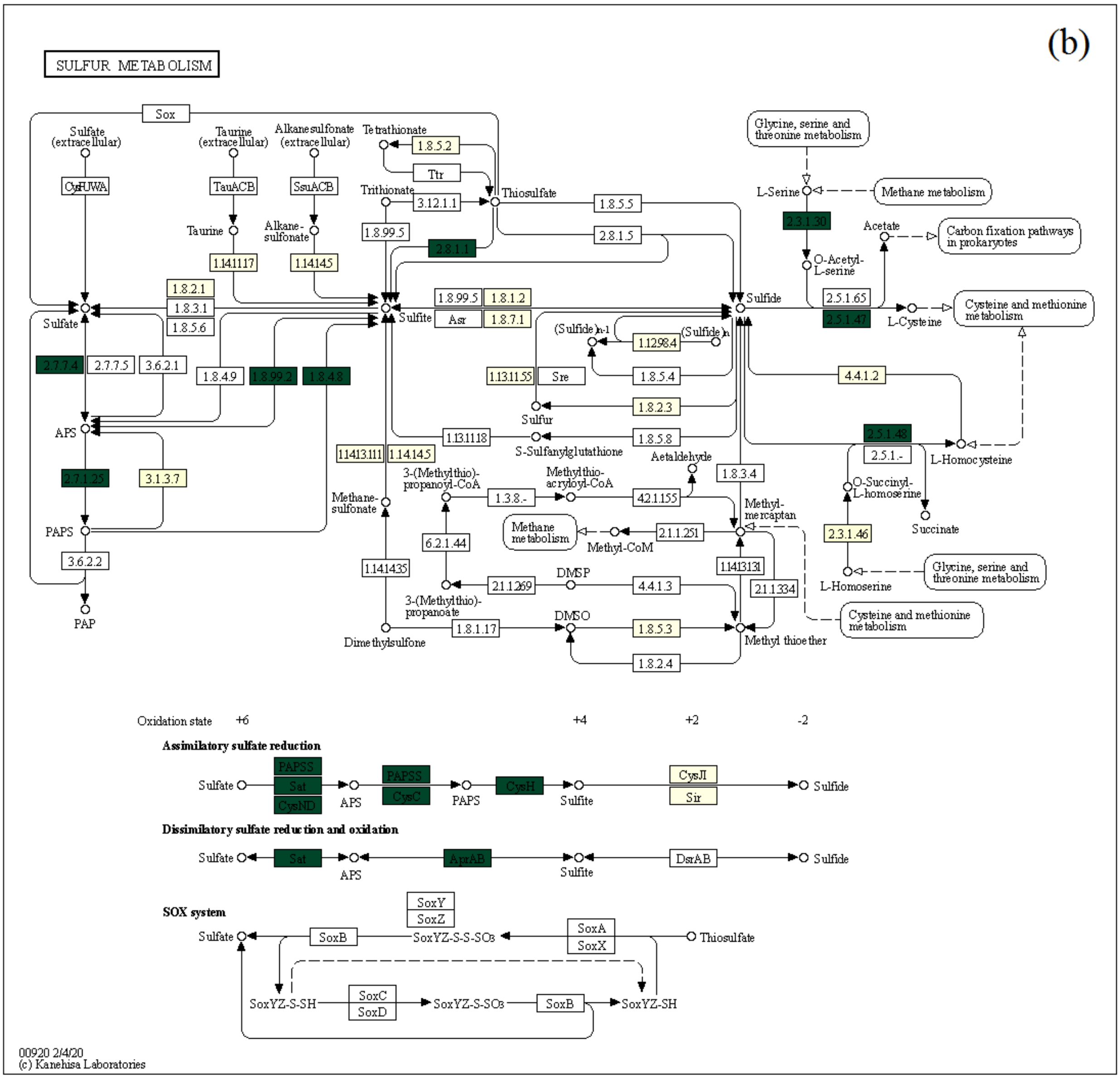

(b)
SULFUR METABOLISM
Sox
Sulfate (extracellular)
CysPUWA
Taurine (extracellular)
TauACB
Alkanesulfonate (extracellular)
SsuACB
Tetrathionate
1.8.5.2
Ttr
Trithionate
3.12.1.1
Thiosulfate
1.8.5.5
2.8.1.5
1.8.99.5
2.8.1.1
Taurine
Alkane-sulfonate
1.14.11.17
1.14.14.5
1.8.2.1
1.8.3.1
1.8.5.6
Sulfate
Sulfite
1.8.99.5
1.8.1.2
Asr
1.8.7.1
Sulfide
2.7.7.4
2.7.7.5
3.6.2.1
1.8.4.9
1.8.99.2
1.8.4.8
APS
2.7.1.25
3.1.3.7
PAPS
3.6.2.2
PAP
(Sulfide)n-1
(Sulfide)n
1.12.98.4
1.13.11.55
Sre
1.8.5.4
1.8.2.3
Sulfur
1.13.11.18
1.8.5.8
S-Sulfanylglutathione
1.14.13.111
1.14.14.5
Methane-sulfonate
3-(Methylthio)-propanoyl-CoA
1.3.8.-
Methylthio-acryloyl-CoA
4.2.1.155
Aetaldehyde
1.8.3.4
Methyl-mercaptan
6.2.1.44
Methane metabolism
Methyl-CoM
2.1.1.251
1.14.14.35
DMSP
2.1.1.269
4.4.1.3
1.14.13.131
2.1.1.334
3-(Methylthio)-propanoate
DMSO
1.8.1.17
1.8.5.3
Methyl thioether
Dimethylsulfone
1.8.2.4
Glycine, serine and threonine metabolism
L-Serine
Methane metabolism
2.3.1.30
Acetate
Carbon fixation pathways in prokaryotes
O-Acetyl-L-serine
2.5.1.65
2.5.1.47
L-Cysteine
Cysteine and methionine metabolism
4.4.1.2
2.5.1.48
2.5.1.-
L-Homocysteine
O-Succinyl-L-homoserine
2.3.1.46
Succinate
L-Homoserine
Glycine, serine and threonine metabolism
Cysteine and methionine metabolism
Oxidation state +6 +4 +2 -2
Assimilatory sulfate reduction
Sulfate
PAPSS
Sat
CysND
APS
PAPSS
CysC
PAPS
CysH
Sulfite
CysJI
Sir
Sulfide
Dissimilatory sulfate reduction and oxidation
Sulfate
Sat
APS
AprAB
Sulfite
DsrAB
Sulfide
SOX system
Sulfate
SoxB
SoxY
SoxZ
SoxYZ-S-S-SO3
SoxA
SoxX
Thiosulfate
SoxYZ-S-SH
SoxC
SoxD
SoxYZ-S-SO3
SoxB
SoxYZ-SH
00920 2/4/20
(c) Kanehisa Laboratories

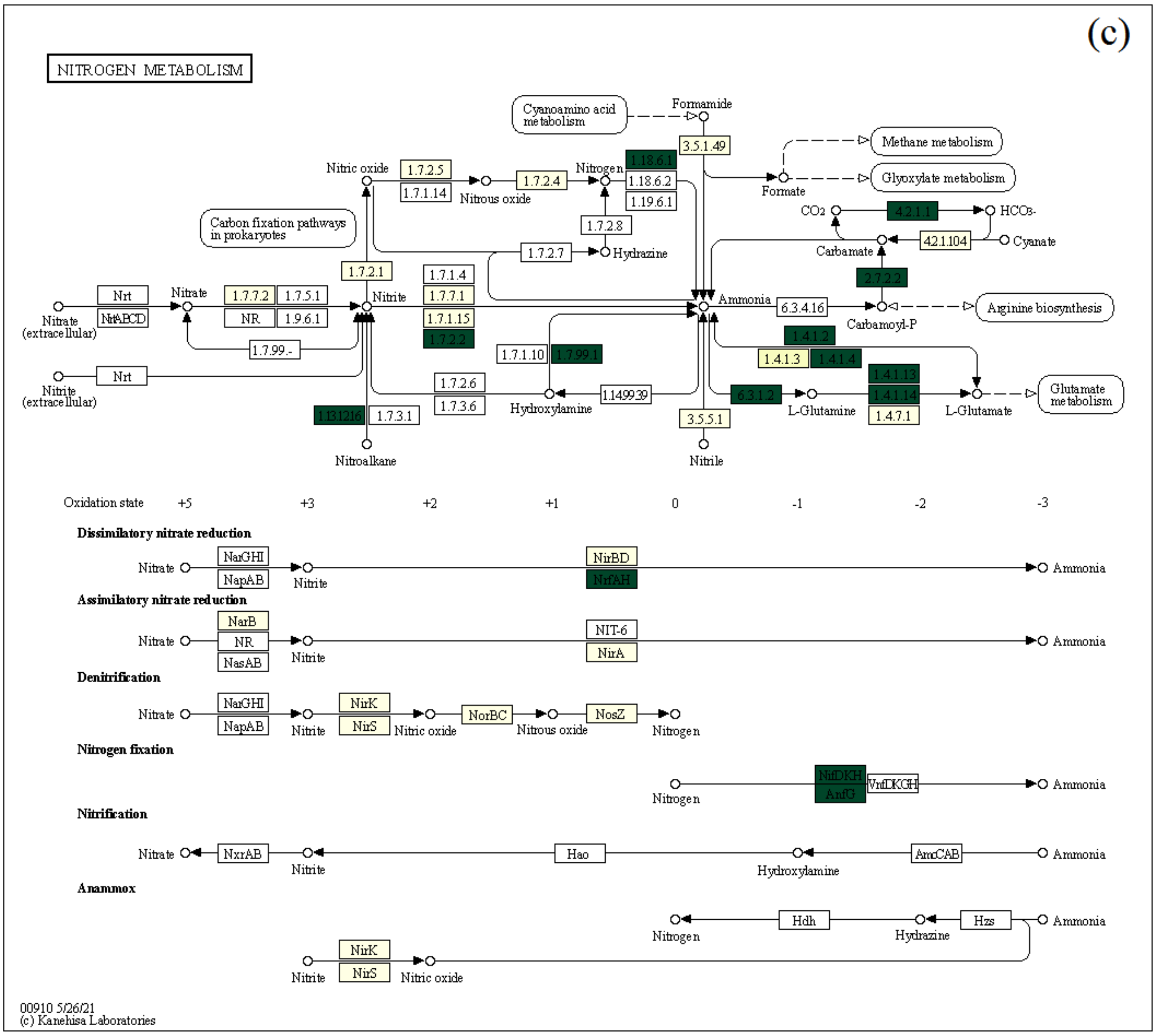

Figure S7. Predicted enzyme profile for “Methane metabolism” (a), “Sulfur metabolism” (b), and “Nitrogen metabolism” (c) pathways of microorganisms in the water sample from production well 7062. The graphs were generated with KEGG Color mapper tool (http://www.genome.jp/kegg/tool/map_pathway3.html) using the Color Mapping files generated by Local Mapper module of iVikodak. Color intensity indicates an average gene (enzyme) proportion in sample. The pathways probably employed by microorganisms in the sample are marked by red.

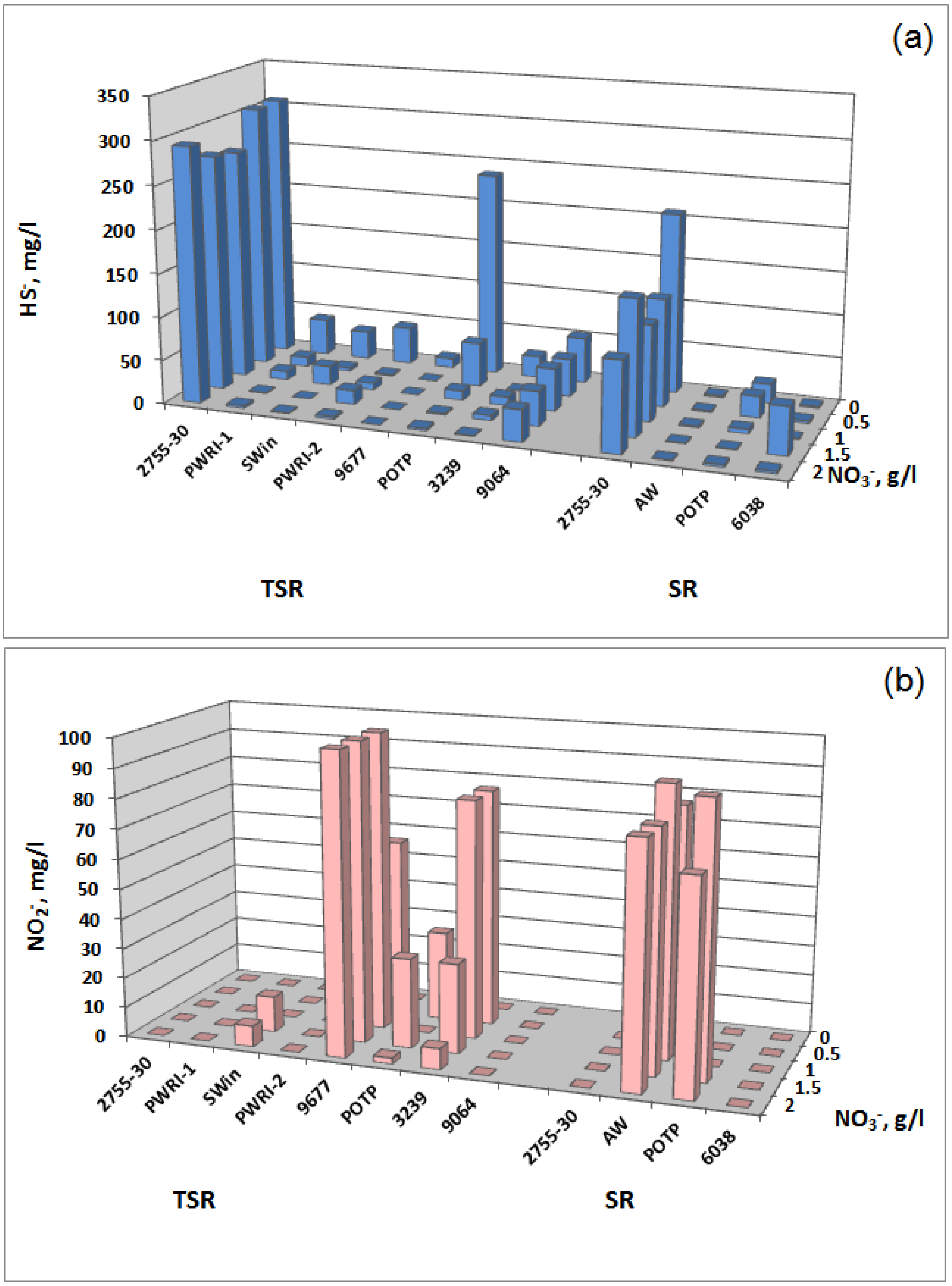

Figure S8. Production of sulfide (a) and nitrite (b) by thiosulfate-reducing (TSR) and sulfate-reducing (SR) bacteria of injection and production water samples in the media with different initial nitrate concentrations. Enrichments 2755-30, PWRI-1, PWRI-2, and POTP were incubated at 42 °C; enrichments 9677, 3239, AW, and 6038 were incubated at 55 °C Cultivation duration was 30 days.

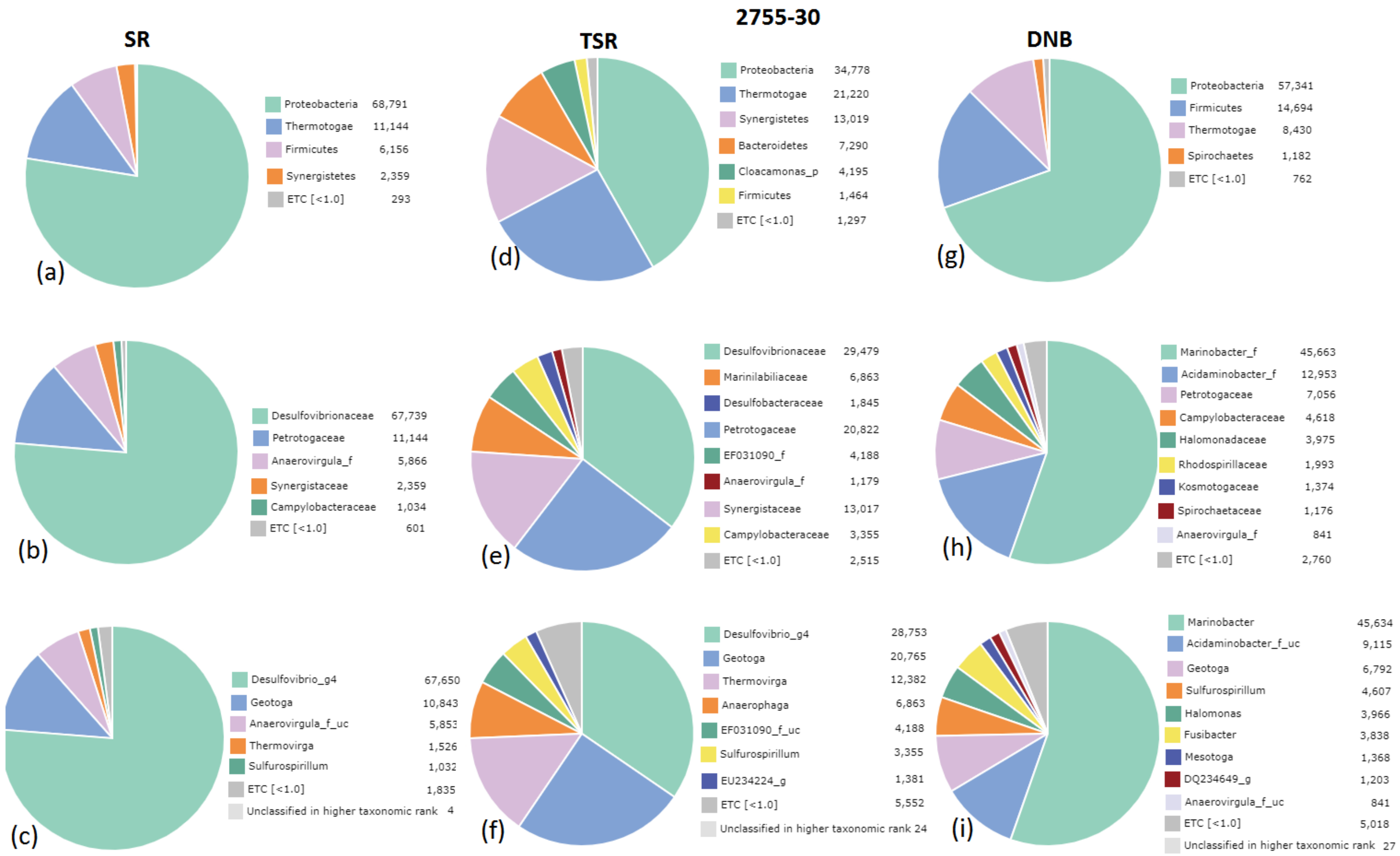
2755-30
SR
TSR
DNB
Proteobacteria 68,791
Thermotogae 11,144
Firmicutes 6,156
Synergistetes 2,359
ETC [<1.0] 293
(a)
Proteobacteria 34,778
Thermotogae 21,220
Synergistetes 13,019
Bacteroidetes 7,290
Cloacamonas_p 4,195
Firmicutes 1,464
ETC [<1.0] 1,297
(d)
Proteobacteria 57,341
Firmicutes 14,694
Thermotogae 8,430
Spirochaetes 1,182
ETC [<1.0] 762
(g)
Desulfovibrionaceae 67,739
Petrotogaceae 11,144
Anaerovirgula_f 5,866
Synergistaceae 2,359
Campylobacteraceae 1,034
ETC [<1.0] 601
(b)
Desulfovibrionaceae 29,479
Marinilabiliaceae 6,863
Desulfobacteraceae 1,845
Petrotogaceae 20,822
EF031090_f 4,188
Anaerovirgula_f 1,179
Synergistaceae 13,017
Campylobacteraceae 3,355
ETC [<1.0] 2,515
(e)
Marinobacter_f 45,663
Acidaminobacter_f 12,953
Petrotogaceae 7,056
Campylobacteraceae 4,618
Halomonadaceae 3,975
Rhodospirillaceae 1,993
Kosmotogaceae 1,374
Spirochaetaceae 1,176
Anaerovirgula_f 841
ETC [<1.0] 2,760
(h)
Desulfovibrio_g4 67,650
Geotoga 10,843
Anaerovirgula_f_uc 5,853
Thermovirga 1,526
Sulfurospirillum 1,032
ETC [<1.0] 1,835
Unclassified in higher taxonomic rank 4
(c)
Desulfovibrio_g4 28,753
Geotoga 20,765
Thermovirga 12,382
Anaerophaga 6,863
EF031090_f_uc 4,188
Sulfurospirillum 3,355
EU234224_g 1,381
ETC [<1.0] 5,552
Unclassified in higher taxonomic rank 24
(f)
Marinobacter 45,634
Acidaminobacter_f_uc 9,115
Geotoga 6,792
Sulfurospirillum 4,607
Halomonas 3,966
Fusibacter 3,838
Mesotoga 1,368
DQ234649_g 1,203
Anaerovirgula_f_uc 841
ETC [<1.0] 5,018
Unclassified in higher taxonomic rank 27
(i)

(**A**)

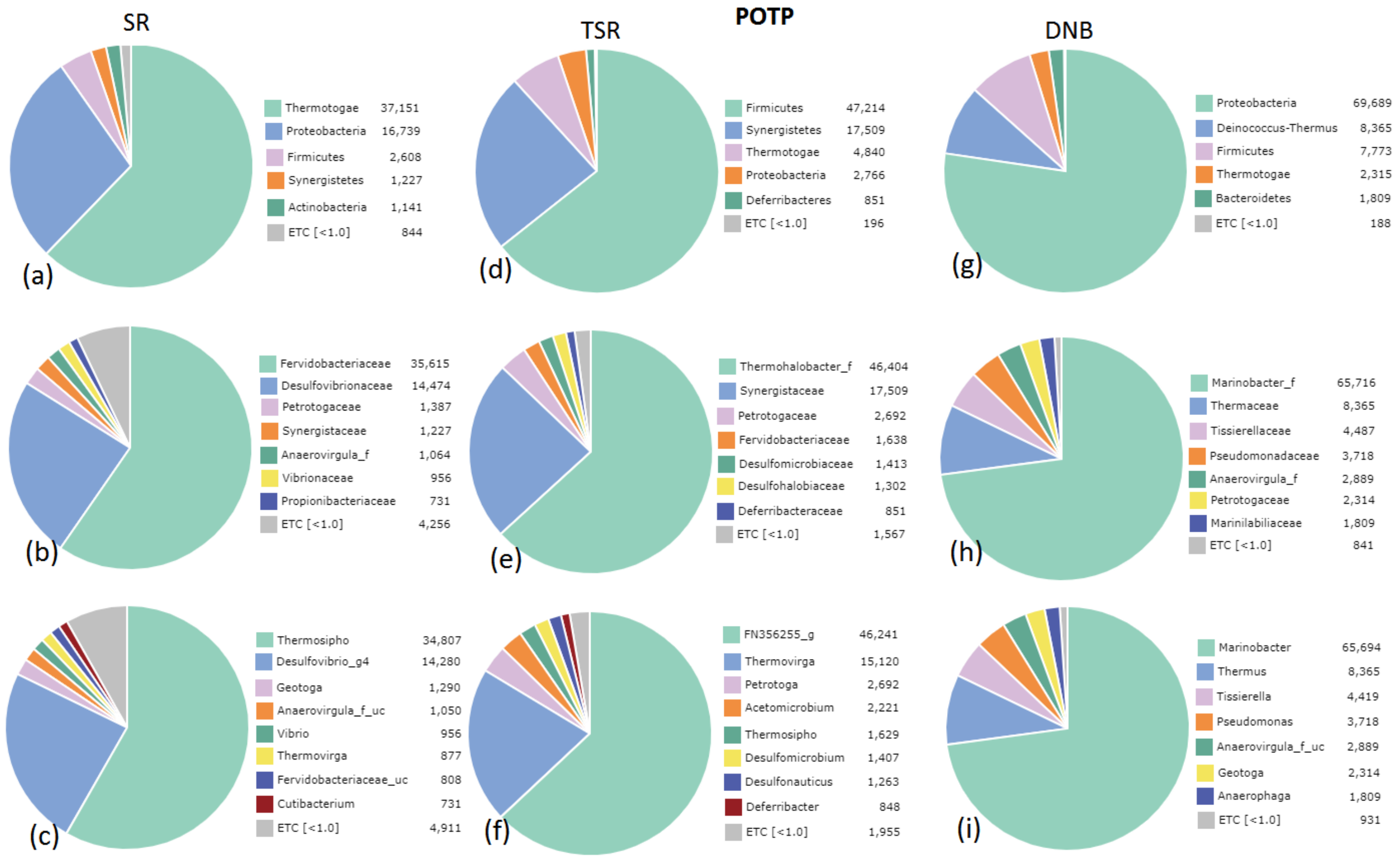

(**B**)

Figure S9. The numbers of sequences in the libraries and bacterial diversity in sulfate-reducing (SR), thiosulfate-reducing (TSR), and denitrifying (DNB) enrichments obtained from the near-bottom zone of injection well 2755 (30 m^3) (A) and from petroleum oil treatment plant (POTP) (B) at the phylum (a, d, g), family (b, e, h), and genus (c, f, i) levels based on the 16S rRNA sequence analysis. The total numbers of sequences in libraries of SR, TSR and DNB enrichments from 2755-30 sample are 88743, 83263, and 82409, and from POTP sample are 59710, 73376, and 90139, respectively.

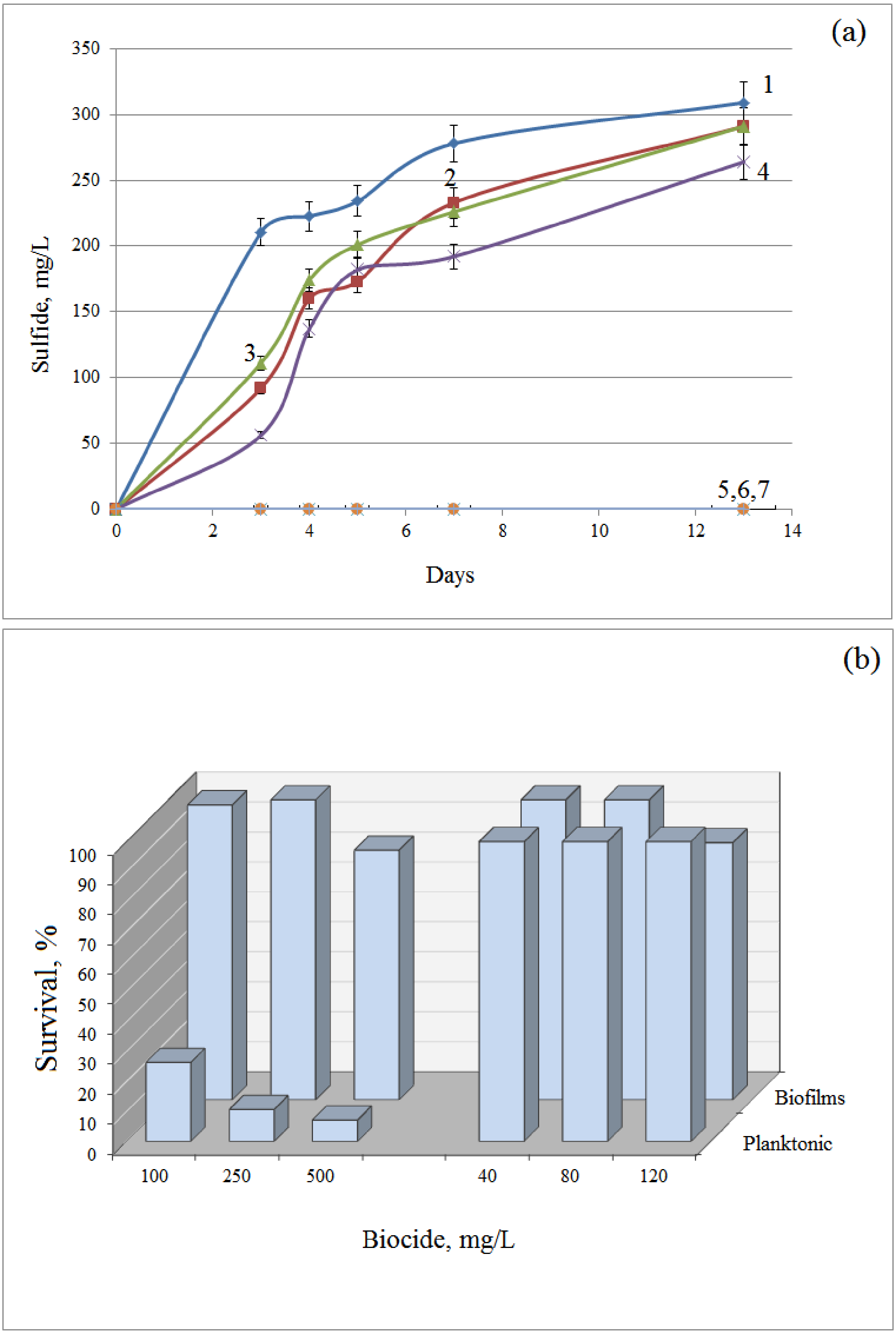

Figure S10. Effect of glutaraldehyde and Rancid on sulfide production (a) and survival (b) of sulfate-reducing bacterium *D. alaskensis* strain Kaz19 in planktonic form and biofilms. Designations in figure (a): Control medium without biocides (Line 1) and with Rancid at concentration 40 (Line 2), 80 (Line 3), and 120 mg·L^{-1} (Line 4) and with glutaraldehyde at concentrations 100 (Line 5), 250 (Line 6), and 500 mg·L^{-1} (Line 7).
